# Intersectionality in AI and Machine Learning for Health Care: Protocol for a Scoping Review

**DOI:** 10.2196/103601

**Published:** 2026-09-30

**Authors:** Duy A Dinh, Julia St Louis, Erin Ziegler, Patricia Gongal, Katie L Bain, Ani Orchanian-Cheff, Sandra Holdsworth, Shilpa Raju, Divya Sharma, Mamatha Bhat, Kathleen A. Sheehan, Suze Berkhout

**Affiliations:** 1 Faculty of Medicine University of Toronto Toronto, ON Canada; 2 Lawrence S. Bloomberg Faculty of Nursing University of Toronto Toronto, ON Canada; 3 Daphne Cockwell School of Nursing Toronto Metropolitan University Toronto, ON Canada; 4 University of Alberta Edmonton, AB Canada; 5 Canadian Donation and Transplantation Research Program Edmonton, AB Canada; 6 Library and Information Services University Health Network Toronto, ON Canada; 7 Department of Mathematics and Statistics York University Toronto, ON Canada; 8 Department of Biostatistics Dalla Lana School of Public Health University of Toronto Toronto, ON Canada; 9 Ajmera Transplant Program University Health Network Toronto, ON Canada; 10 Division of Gastroenterology Faculty of Medicine University of Toronto Toronto, ON Canada; 11 Toronto General Hospital Research Institute University Health Network Toronto, ON Canada; 12 Department of Psychiatry University of Toronto Toronto, ON Canada; 13 Centre for Mental Health University Health Network Toronto, ON Canada

**Keywords:** artificial intelligence, AI, machine learning, intersectionality, equity, health care

## Abstract

**Background:**

AI and machine learning (ML) are increasingly being used in health care settings but may reproduce or exacerbate systemic biases. The integration of intersectionality and equity-related concepts offers an analytical framework to guide more reflexive, equity-oriented AI and ML design and implementation, particularly when paired with a coproduction philosophy that meaningfully includes community-based collaborators and knowledge users. However, there is limited synthesis on how intersectionality is conceptualized and operationalized in this context, and the extent to which interdisciplinary collaborators and patient partners are involved in such undertakings is unknown.

**Objective:**

This study aims to synthesize how intersectionality is conceptualized and operationalized, including frameworks, pedagogical tools, and methods, in AI and ML for health care, as well as the extent to which such projects use participatory or coproduction frameworks.

**Methods:**

The proposed scoping review will be conducted in accordance with the scoping review framework developed by the Joanna Briggs Institute. With the assistance of a research librarian, the following databases will be searched for published articles with primary data: MEDLINE, Embase, Emcare Nursing, APA PsycInfo, Cochrane Database of Systematic Reviews, and Cochrane Central Register of Controlled Trials. Eligible studies will include any primary article that applies intersectionality in guiding the design and implementation of AI and ML in health care. This review will only include studies written in English. Two independent reviewers will screen the title and abstracts of articles, followed by its full-text review, for eligibility against a priori inclusion criteria. Conflicts regarding inclusion or exclusion will be resolved through consensus. Data will be extracted from the included studies and summarized narratively, supplemented by tables and charts. Patient or family partners will be engaged throughout the review process to refine the scope of the review, interpret findings, and support knowledge translation efforts, ensuring outputs are equity oriented and responsive to community priorities.

**Results:**

Preliminary searches yielded a total of 4191 records across 6 databases. The scoping review will be completed by October 2026.

**Conclusions:**

This scoping review will map how intersectionality is used in AI and ML research in health care, with a particular focus on how the term is conceptualized, operationalized, and used in the context of community participatory practice. Findings from the review will identify key gaps in the literature and provide community-relevant recommendations on how to meaningfully integrate intersectionality into the development of AI and ML for health care.

**International Registered Report Identifier (IRRID):**

DERR1-10.2196/103601

## Introduction

### Background

AI and machine learning (ML) are increasingly being applied in health care settings [1-4]. These tools are used in a variety of clinical and public health applications, including diagnosis, risk prediction, triage, and treatment planning [5]. As AI and ML become embedded in routine clinical decision-making, considerations of their trustworthiness and influence on power and resource distribution within health systems have become more pressing [5].

Despite their proposed benefits for individual health outcomes and system-level efficiency, these technologies may encode algorithmic biases, exacerbating inequities among marginalized communities [6]. AI models trained without consideration of social contexts may reflect systemic biases, highlighting the need for more inclusive training approaches and contextual data to enhance the equity of AI models [7]. Equity in AI requires treating identity as more than a sequence of independent variables (eg, race, gender, class, and disability). It requires recognizing the overlapping and dynamic nature of social identities, as well as the influence of broader sociostructural factors (eg, colonialism, policy, and cisheteronormativity).

Intersectionality is an analytical framework that examines how interconnected and dynamic systems of power and social identities influence the lived experiences of individuals and communities [8]. The framework has been leveraged to mitigate systemic biases in health-related AI and ML applications (eg, dermatology machine learning models, opioid treatment, and breast cancer screening models) by moving beyond individual subgroup comparisons and examining how model performance, access, or risk differs across intersecting social positions, including race or ethnicity, sex, age, socioeconomic status, geography, and care context [9-15]. As such, the application of an intersectional framework has been proposed as a key method in promoting equitable AI and ML in the health sciences [7,16-22].

A recent review of intersectional perspectives in AI and ML guidelines and frameworks revealed substantial gaps and heterogeneity in how guidance documents address intersectional bias, particularly for underrepresented dimensions such as disability and citizenship [23]. However, the review focused on guidance documents and conceptual frameworks rather than primary studies and did not examine how intersectionality is operationalized in applied AI and ML development or the extent to which community and patient partners are involved. As such, there has been limited evidence synthesis on how intersectionality has been operationalized in practice, including frameworks, pedagogical tools, and methods used, to guide the design and implementation of AI and ML tools in health care. The present review addresses this distinct, nonoverlapping space by specifically mapping primary AI and ML studies that apply intersectionality. Additionally, the inclusion of impacted communities and knowledge users is both promising and vexed as a means of addressing disparate impacts of AI and ML on structurally disadvantaged and equity-denied groups [24]. Beyond the aforementioned review, a search of the MEDLINE and Cochrane Library databases revealed no current literature reviews focusing on intersectionality in the context of AI and ML tools in health care. The paucity of evidence syntheses on this topic necessitates a scoping review.

To that end, our primary objective is to map the literature on how the intersectionality theory is applied in AI and ML for health care. We aim to synthesize existing evidence and highlight gaps in the literature to clarify concepts regarding the research topic, undertaking this analysis within a participatory research framework.

### Review Questions

The primary research question of this scoping review is as follows: How is intersectionality conceptualized and operationalized to guide the design and implementation of AI and ML for health care?

The secondary research questions are as follows: (1) What are the conceptualizations of intersectionality in this context? (2) How are frameworks, pedagogical tools, and methods of intersectionality and intersectionality-related concepts operationalized in the literature? (3) What are the specific factors or characteristics used in the conceptualization or operationalization of intersectionality? (4) Where along the AI and ML development life cycle is intersectionality applied? and (5) What participatory approaches are used within this body of research?

## Methods

### Overview

The proposed scoping review will be conducted in accordance with the scoping review framework developed by the Joanna Briggs Institute (JBI) [25], originally described by Arksey and O’Malley [26]. The proposed scoping review includes the following stages: (1) identifying the research questions; (2) information sources and search strategy; (3) study selection; (4) data extraction and synthesis; and (5) collating, summarizing, and reporting the results, with patient partner participation at every stage. This scoping review protocol is registered with the Open Science Framework [27].

### Knowledge User and Community Engagement

Knowledge users (KUs) and community engagement are central to ensuring reviews produce evidence syntheses that are relevant and responsive to stakeholders most affected by AI and ML in health care [28,29]. Meaningful engagement with patient partners, AI and ML researchers, and engineers is necessary to mitigate misinterpretation of priorities in AI and ML development, build trust, and support recommendations that are relevant and salient to patients and community stakeholders. The present scoping review will prioritize and integrate the opinions of patients with lived experience of solid organ transplantation (authors SR and SH), recruited from the Canadian Donation and Transplantation Research Program’s patient, family, and donor platform, representing meaningful coproduction [29]. Specifically, patient partners will be engaged in (1) reviewing and shaping the protocol, including refining the scope of the review; (2) identifying relevant outcomes and variables; (3) interpreting and contextualizing extracted data; (4) drafting and reviewing the final manuscript; and (5) knowledge translation activities, including translating key findings into accessible outputs for patients and communities [28,29].

### Search Strategy

With the assistance of a research librarian, the search strategy will aim to identify published studies on the topic.

A comprehensive search strategy was developed using a combination of database-specific subject headings and text words for the main concepts of AI and intersectionality (Multimedia Appendix 1). Searches were limited to studies published in English. Conference materials were removed from the Embase and Emcare databases. The following databases were searched on January 26, 2026: MEDLINE, Embase, Emcare Nursing, APA PsycInfo, Cochrane Database of Systematic Reviews, and Cochrane Central Register of Controlled Trials. The reference lists of the included publications will also be searched and considered for inclusion.

### Eligibility Criteria

Eligible studies will provide insight into the use of intersectionality in guiding the design and implementation of AI and ML for health care, including but not limited to applications pertaining to clinical care, public health, health systems, health services, and health-related research contexts. For the purposes of this review, AI and ML are defined broadly as computer programs that use algorithms and statistical models to learn or identify patterns from data to generate predictions, classifications, recommendations, decisions, or new content [30,31]. These include supervised, unsupervised, and reinforcement learning; neural networks and deep learning; natural language processing, including large language and generative models; and other data-trained statistical or clinical prediction models using ML methods. Studies that do not use AI or ML or do not apply intersectionality and intersectionality-related concepts will be excluded. This review will include studies from all contexts, regardless of country or region and date of publication.

The proposed study eligibility criteria are presented in Textbox 1.

**Study eligibility criteria.**

**Inclusion criteria**

- Published at any time
- Published in any country
- Peer-reviewed research article with primary quantitative or qualitative data
- Focused on intersectionality in AI and machine learning (ML) in health care

**Exclusion criteria**

- Studies not published in English
- Studies lacking peer-review and/or primary research data (ie, conference abstracts, dissertations, literature reviews, commentaries, editorials, and opinions)
- Studies that do not apply intersectionality theory or intersectionality-related concepts
- Studies not focused on AI or ML
- Studies not focused on health care or health-related topics

### Types of Sources

This scoping review will consider all the literature contributing primary qualitative or quantitative data on intersectionality in AI and ML health care applications. The review will consider, but will not be limited to, experimental and quasi-experimental studies (eg, randomized and nonrandomized controlled trials), analytical observational studies (eg, retrospective cohort studies and analytical cross-sectional studies), and descriptive observational studies (eg, case series). To prevent duplication of data, secondary sources of information, including systematic reviews and opinion papers, will not be considered. While the conceptualizations of intersectionality frequently appear in secondary sources, the primary objective of this review is to map how intersectionality is operationalized in applied AI and ML development. As such, we restrict eligibility to primary studies to maintain this focus on applied AI and ML tools and to avoid double-counting evidence already captured in existing reviews of frameworks and guidance. Only studies in English will be considered.

### Study or Source of Evidence Selection

All identified citations will be collated and uploaded into the systematic review software Covidence (Veritas Health Innovation), and duplicates will be removed. Prior to screening, the research team will conduct meetings and pilot testing to ensure consistency and reliability in the screening process. Following the framework described in the *JBI Manual for Evidence Synthesis* [25], a random sample of 25 titles and abstracts will be assessed by 2 reviewers (DD, JSL, and/or EZ) against the inclusion criteria for the review. Interrater reliability will be measured, and conflicts will be discussed with additional team members (SB, SR, and SH) to achieve consensus. The eligibility criteria will be modified as necessary. The pilot test will be repeated until a Cohen κ of ≥0.80, indicating strong or almost perfect agreement, is achieved [32]. Following pilot testing, the reviewers will begin the screening process, and regular meetings will be held to discuss any discrepancies and ensure consensus.

Two reviewers will then assess the full text of selected citations in detail against the inclusion criteria (DD, JSL, and/or EZ). Reasons for exclusion of sources of evidence at the full-text screening stage that do not meet the inclusion criteria will be recorded and reported in the scoping review. Any disagreements that arise between the reviewers at this stage of the selection process will be resolved through discussion and, if necessary, consultation with additional reviewers (SB, SR, and SH). The search results and the study inclusion process will be reported in full in the final scoping review and presented in a PRISMA-ScR (Preferred Reporting Items for Systematic Reviews and Meta-Analyses Extension for Scoping Reviews) flow diagram [33].

### Data Extraction

Data will be extracted from the included studies using a data extraction sheet by 2 independent reviewers (DD, JSL, and/or EZ). Particular attention will be given to fields related to the conceptualization of intersectionality. The data extraction tool was developed by all reviewers in collaboration with patient partners. The data will be verified by an additional reviewer (SB). The data extracted will include variables but are not limited to the following: study characteristics and methods (ie, author, year, country or region of study, sample size, target population, study design, description of study or intervention methodology, study objectives, health care topic or field of interest, and disciplinary background), AI- and ML-related data (ie, model type or algorithm family, data modality [eg, tabular, image, text, or multimodal], and clinical task [eg, prediction, diagnosis, triage, or screening]), intersectionality-related data (ie, definitions or conceptualizations of intersectionality; definitions or conceptualizations of intersectionality-related terms; operationalization of intersectionality*;* description of intersectionality-related frameworks, if applicable; description of intersectionality-related visual tools, if applicable; description of intersectionality-related pedagogical tools, if applicable; description of intersectionality-related methods, if applicable; factors or characteristics used in conceptualization or operationalizations of intersectionality; and the stage at which intersectionality is applied in AI and ML or research development), and community member and partner participation (ie, description of involvement of KUs, community members, and/or patient partners, if applicable). The draft data extraction sheets will undergo iterative modifications and revisions, as necessary, throughout the data extraction process. Modifications will be detailed in the final scoping review.

### Data Analysis and Presentation

Data analyses and syntheses will be guided by team discussions, including with our patient partners, to ensure relevant and sensitive conclusions [28,29]. A narrative summary will be produced to describe and summarize the extracted data. In addition, included studies will be grouped according to how intersectionality is engaged: conceptual framing only, methodological operationalization, participatory or coproduction application, implementation or governance application, or mixed approaches. Tables and charts, including tables summarizing the included studies and conceptualizations and operationalizations of intersectionality in AI and ML for health care, will accompany the narrative summary. The results of this scoping review will be reported following the PRISMA-ScR guidelines [33].

### Dissemination Plan

Findings will be disseminated through peer-reviewed publication and conference presentations and reported in accordance with PRISMA-ScR. In keeping with our coproduction approach, patient and community partners will collaborate on plain-language summaries and other accessible knowledge translation outputs tailored to patient, community, and knowledge user audiences.

## Results

The scoping review process began in January 2026. Database searches were conducted on January 26, 2026. As of January 26, 2026, the search yielded 4191 records across databases: 1063 records from MEDLINE, 1254 records from Embase, 993 records from Emcare, 869 records from PsycInfo, 12 records from Cochrane Central Register of Controlled Trials, and 0 records from Cochrane Database of Systematic Reviews. Title and abstract screening of articles will occur in April 2026, full-text screening will be done in May 2026, and data extraction and analysis will be completed between June 2026 and August 2026. We aim to submit the results for publication by the end of October 2026. Results from the inclusion and exclusion screening process will be reported using the PRISMA-ScR flowchart; preliminary findings are shown in Figure 1.

**Figure 1:**
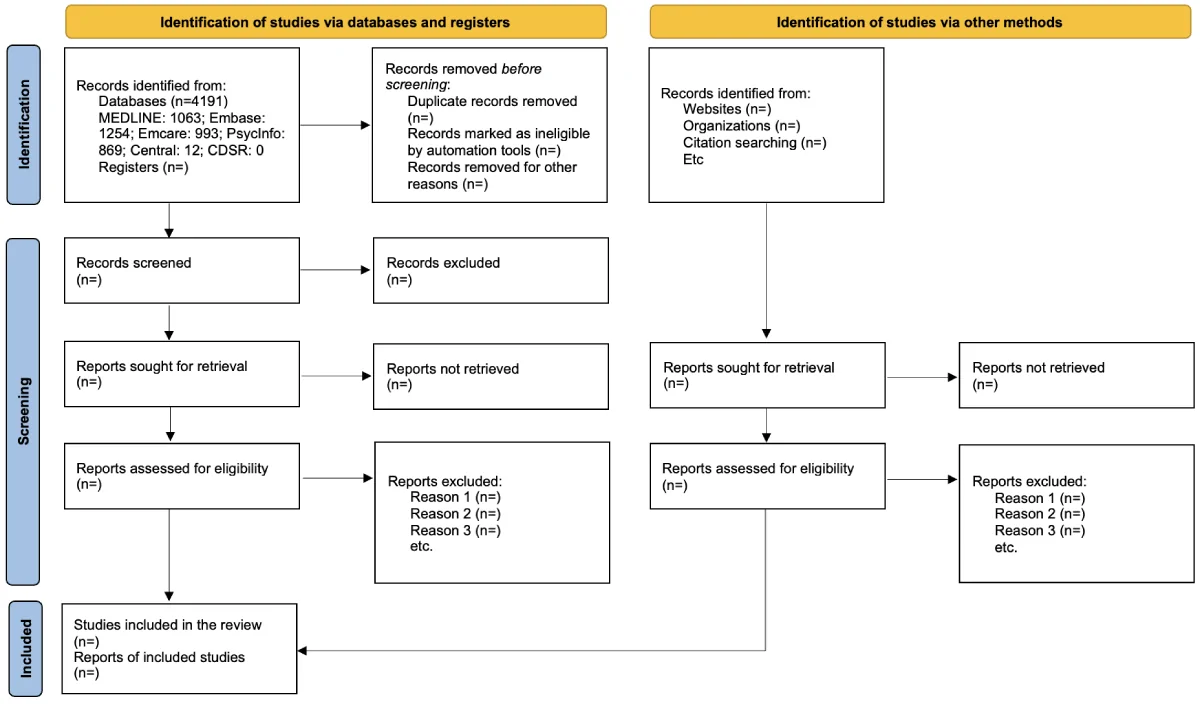
PRISMA-ScR (Preferred Reporting Items for Systematic Reviews and Meta-Analyses Extension for Scoping Reviews) flowchart showing preliminary findings as of January 26, 2026.

## Discussion

### Principal Findings

The present scoping review will comprehensively map how intersectionality is conceptualized and operationalized in AI and ML in health care. Although intersectionality and equity-related concepts are increasingly being raised in conversations about fair AI and ML, it remains unclear how the framework is conceptualized and operationalized [7,16-19]. Importantly, in AI and ML contexts, intersectionality extends beyond reporting model performance across isolated demographic subgroups alone; rather, it involves attention to overlapping identities, structural conditions, and systems of power. This review is expected to identify and synthesize how intersectionality is used across the literature, including the frameworks, tools, and methods that support its application, as well as the extent to which KUs are involved. We anticipate heterogeneity in how intersectionality is operationalized across primary studies, including variation in what and how many intersecting factors are considered, ranging from demographic characteristics to broader systemic conditions such as stigma. We aim to emphasize the role of integration of intersectionality to guide more reflexive, equity-oriented AI and ML design and implementation, particularly when paired with coproduction approaches. Results from the review will help clarify current practices, identify gaps in the literature, and inform future efforts to develop more equitable and fair AI and ML health care applications.

### Comparisons to Prior Work

As introduced previously, existing reviews have examined the use of intersectionality in AI and ML in guidance documents and conceptual frameworks, mapping how such documents address intersectional biases across the AI life cycle [23]. Our review will build upon this, focusing on primary studies that apply intersectionality in the design and implementation of AI and ML tools for health care. In doing so, we anticipate clarifying not only how intersectionality is conceptualized but also how it is actually operationalized in applied AI and ML tools in health care.

Additionally, the proposed scoping review places emphasis on participatory methods. Cocreation with patient partners will take place at all phases of the scoping review process to align priorities and ensure recommendations are salient to patient and KU stakeholders.

### Limitations

The present protocol must be considered in light of several limitations. First, although the search strategy was designed to be broad and systematic, some records may still be missed because of disciplinary heterogeneity in terminology, particularly regarding intersectionality and other equity-related concepts. To address this, the research team and librarian engaged in in-depth discussions to consider a diversity of related terms to ensure breadth in the search strategy. Second, the review will only consider English-language records and primary literature, which may narrow the scope of included evidence. Finally, consistent with best-practice scoping review methodology [26], the scope of the data will be mapped descriptively without assessment of the source’s quality.

### Conclusions

This scoping review protocol outlines the rationale and methods for mapping how intersectionality is used in AI and ML research in health care, with a particular focus on how the term is conceptualized, operationalized, and used in the context of community participatory practice. Findings from the review will identify key gaps in the literature and provide community-relevant recommendations on how to meaningfully integrate intersectionality into the development of AI and ML for health care.

## Appendix

Multimedia Appendix 1 Comprehensive search strategy.

## Abbreviations

JBI: Joanna Briggs Institute
KU: knowledge user
ML: machine learning
PRISMA-ScR: Preferred Reporting Items for Systematic Reviews and Meta-Analyses Extension for Scoping Reviews
