# Supplementary material for: Intersectionality in AI and Machine Learning for Health Care: Protocol for a Scoping Review
Source: JMIR Res Protoc. 2026 Sep 30;15:e103601. doi: 10.2196/103601 (PMC13669704; doi:10.2196/103601)
Supplement: Supplementary file 1 [file resprot_v15i1e103601_app1.docx]

**Appendix I**

**Search strategies as run on January 26, 2026**

Ovid MEDLINE(R) ALL <1946 to January 23, 2026>

| **#** | **Searches** | **Results** | **Type** | **Runtime** |  |  |
| --- | --- | --- | --- | --- | --- | --- |
|  | | | | | | |
| 1 | exp Artificial Intelligence/ | 267744 | Advanced | 0.51 |  |  |
| 2 | Big Data/ | 3531 | Advanced | 0.07 |  |  |
| 3 | Biomedical Technology/ | 7602 | Advanced | 0.03 |  |  |
| 4 | Computer Heuristics/ | 53 | Advanced | 0.03 |  |  |
| 5 | Computer-Aided Design/ | 20259 | Advanced | 0.00 |  |  |
| 6 | Data Aggregation/ | 79 | Advanced | 0.03 |  |  |
| 7 | Data Analysis/ | 5157 | Advanced | 0.00 |  |  |
| 8 | Data Mining/ | 12314 | Advanced | 0.00 |  |  |
| 9 | Data Science/ | 1038 | Advanced | 0.00 |  |  |
| 10 | Decision Making, Computer-Assisted/ | 2895 | Advanced | 0.02 |  |  |
| 11 | Decision Trees/ | 13009 | Advanced | 0.00 |  |  |
| 12 | Deep Learning/ | 36465 | Advanced | 0.00 |  |  |
| 13 | exp Automation/ | 76453 | Advanced | 0.12 |  |  |
| 14 | Expert Systems/ | 3529 | Advanced | 0.03 |  |  |
| 15 | Image Interpretation, Computer-Assisted/ | 51663 | Advanced | 0.04 |  |  |
| 16 | Image Processing, Computer-Assisted/ | 154923 | Advanced | 0.00 |  |  |
| 17 | Machine Learning/ | 64991 | Advanced | 0.00 |  |  |
| 18 | Neural Networks, Computer/ | 67844 | Advanced | 0.02 |  |  |
| 19 | Numerical Analysis, Computer-Assisted/ | 5821 | Advanced | 0.01 |  |  |
| 20 | Pattern Recognition, Automated/ | 27132 | Advanced | 0.01 |  |  |
| 21 | Radiographic Image Interpretation, Computer-Assisted/ | 17718 | Advanced | 0.02 |  |  |
| 22 | Signal Processing, Computer-Assisted/ | 54427 | Advanced | 0.02 |  |  |
| 23 | exp Supervised Machine Learning/ | 15878 | Advanced | 0.23 |  |  |
| 24 | Support Vector Machine/ | 12979 | Advanced | 0.00 |  |  |
| 25 | Unsupervised Machine Learning/ | 1376 | Advanced | 0.00 |  |  |
| 26 | "artif#cial intelligence".mp. | 122981 | Advanced | 0.95 |  |  |
| 27 | "big data".mp. | 18827 | Advanced | 0.65 |  |  |
| 28 | ("AI" and intelligence).tw. | 40543 | Advanced | 0.11 |  |  |
| 29 | ((neural* adj2 network*) and computer).mp. | 79677 | Advanced | 2.05 |  |  |
| 30 | ((text? or textual*) adj2 analy*).mp. | 6802 | Advanced | 3.77 |  |  |
| 31 | (algor#th* adj3 (comput* or machine? or intelligen* or art#ficial* or analy*)).mp. | 67190 | Advanced | 5.57 |  |  |
| 32 | (art#ficial* adj2 intelligen*).mp. | 123557 | Advanced | 0.12 |  |  |
| 33 | (automat* adj2 algor#th*).mp. | 8475 | Advanced | 1.37 |  |  |
| 34 | (automat* adj2 pattern? adj2 analy*).mp. | 69 | Advanced | 1.42 |  |  |
| 35 | (automat* adj2 pattern? adj2 recog*).mp. | 27271 | Advanced | 2.46 |  |  |
| 36 | (automat* adj2 pattern? adj2 classifi*).mp. | 26 | Advanced | 1.21 |  |  |
| 37 | (automat*4 and (computer* or technolog*)).mp. | 114204 | Advanced | 1.74 |  |  |
| 38 | (comput* adj1 analy*).mp. | 30329 | Advanced | 0.58 |  |  |
| 39 | (comput* adj2 knowledge adj2 (acquisition or representation*)).mp. | 17 | Advanced | 2.58 |  |  |
| 40 | (comput* adj2 pattern? adj2 analy*).mp. | 84 | Advanced | 0.56 |  |  |
| 41 | (comput* adj2 pattern? adj2 recog*).mp. | 184 | Advanced | 0.25 |  |  |
| 42 | (comput* adj2 pattern? adj2 classifi*).mp. | 7 | Advanced | 0.25 |  |  |
| 43 | (comput* adj2 reasoning*).mp. | 124 | Advanced | 1.82 |  |  |
| 44 | (computat* adj2 intelligen*).mp. | 985 | Advanced | 0.45 |  |  |
| 45 | (computational adj1 approach??).mp. | 18151 | Advanced | 1.22 |  |  |
| 46 | (computational adj1 repurpos*).mp. | 54 | Advanced | 1.82 |  |  |
| 47 | (computer* adj2 vision*).mp. | 13268 | Advanced | 0.63 |  |  |
| 48 | (computer* adj2 heuristic*).mp. | 69 | Advanced | 0.44 |  |  |
| 49 | (data adj1 mining).mp. | 25088 | Advanced | 0.49 |  |  |
| 50 | (deep* adj2 learn*).mp. | 109447 | Advanced | 1.37 |  |  |
| 51 | (deep* adj2 network*).mp. | 22024 | Advanced | 0.14 |  |  |
| 52 | (digital adj2 technolog*).mp. | 14411 | Advanced | 0.25 |  |  |
| 53 | (hierarchic* adj1 learn*).mp. | 293 | Advanced | 0.64 |  |  |
| 54 | (inductive* adj1 machine* adj1 learn*).mp. | 24 | Advanced | 0.95 |  |  |
| 55 | (information?? adj1 technolog*).mp. | 21146 | Advanced | 0.86 |  |  |
| 56 | (knowledge acqui* and computer*).mp. | 533 | Advanced | 1.14 |  |  |
| 57 | (knowledge discovery and computer).mp. | 309 | Advanced | 0.38 |  |  |
| 58 | (knowledge represent* and computer).mp. | 480 | Advanced | 2.55 |  |  |
| 59 | (learning adj1 model* adj1 algorithm*).mp. | 28 | Advanced | 2.37 |  |  |
| 60 | (learning adj2 algorithm*).mp. | 44098 | Advanced | 0.19 |  |  |
| 61 | (learning adj3 labeled data).mp. | 127 | Advanced | 0.55 |  |  |
| 62 | ((logistic* or logit) adj2 regress*).mp. | 543699 | Advanced | 2.81 |  |  |
| 63 | (machine? adj2 assist* adj2 discover*).mp. | 35 | Advanced | 1.69 |  |  |
| 64 | (machine? adj2 intelligen*).mp. | 3840 | Advanced | 0.08 |  |  |
| 65 | (machine? adj2 learn*).mp. | 191739 | Advanced | 0.17 |  |  |
| 66 | (ML adj2 (framework? or tool*)).mp. | 1280 | Advanced | 1.89 |  |  |
| 67 | ML algorithm*.mp. | 6097 | Advanced | 0.18 |  |  |
| 68 | ML application*.mp. | 639 | Advanced | 1.25 |  |  |
| 69 | ML method*.mp. | 3561 | Advanced | 2.27 |  |  |
| 70 | MLP.mp. | 6033 | Advanced | 0.10 |  |  |
| 71 | (native adj2 bayes).mp. | 4 | Advanced | 0.39 |  |  |
| 72 | (learn* adj2 bayes).mp. | 82 | Advanced | 0.16 |  |  |
| 73 | (neural* adj2 network* adj2 (model* or comput*)).mp. | 78013 | Advanced | 1.21 |  |  |
| 74 | (predictive adj2 algor#th*).mp. | 2331 | Advanced | 0.50 |  |  |
| 75 | (predictive adj2 analy*).mp. | 10021 | Advanced | 0.59 |  |  |
| 76 | (random* adj2 forest?).mp. | 44552 | Advanced | 3.31 |  |  |
| 77 | (regulari* adj3 algorithm*).mp. | 794 | Advanced | 1.79 |  |  |
| 78 | (regress* adj2 tree?).mp. | 7427 | Advanced | 0.49 |  |  |
| 79 | (ridge adj2 regress*).mp. | 2286 | Advanced | 0.24 |  |  |
| 80 | RVM.mp. | 982 | Advanced | 0.16 |  |  |
| 81 | (structural equation* adj2 model*).mp. | 42774 | Advanced | 0.98 |  |  |
| 82 | (support adj2 vector).mp. | 42477 | Advanced | 0.38 |  |  |
| 83 | (vector? adj2 machine?).mp. | 39339 | Advanced | 0.41 |  |  |
| 84 | (vector? adj2 network*).mp. | 1017 | Advanced | 0.11 |  |  |
| 85 | adaptive algorithm*.mp. | 899 | Advanced | 0.34 |  |  |
| 86 | art#ficial neural network*.mp. | 23503 | Advanced | 0.75 |  |  |
| 87 | artificial intelligence*.mp. | 123040 | Advanced | 1.31 |  |  |
| 88 | Bayesian network*.mp. | 6871 | Advanced | 0.14 |  |  |
| 89 | big health* data.mp. | 72 | Advanced | 1.63 |  |  |
| 90 | chatgpt*.mp. | 8792 | Advanced | 0.27 |  |  |
| 91 | (classification? adj2 algorithm*).mp. | 7133 | Advanced | 0.54 |  |  |
| 92 | clustering algorithm*.mp. | 6947 | Advanced | 0.20 |  |  |
| 93 | compression algorithm*.mp. | 778 | Advanced | 0.23 |  |  |
| 94 | computational intelligence.mp. | 651 | Advanced | 0.15 |  |  |
| 95 | computer cellular neural network*.mp. | 0 | Advanced | 0.52 |  |  |
| 96 | computer reasoning.mp. | 11 | Advanced | 0.14 |  |  |
| 97 | computer vision system?.mp. | 475 | Advanced | 1.13 |  |  |
| 98 | convolutional neural network*.mp. | 41056 | Advanced | 0.24 |  |  |
| 99 | (crossvalidation or cross-validation).mp. | 41762 | Advanced | 0.55 |  |  |
| 100 | data analytic?.mp. | 4283 | Advanced | 0.70 |  |  |
| 101 | data driven science?.mp. | 96 | Advanced | 0.87 |  |  |
| 102 | data-driven science?.mp. | 96 | Advanced | 0.35 |  |  |
| 103 | deep learning.mp. | 106122 | Advanced | 0.26 |  |  |
| 104 | detection algorithm*.mp. | 7881 | Advanced | 0.26 |  |  |
| 105 | digital intervention?.mp. | 3529 | Advanced | 0.90 |  |  |
| 106 | ensemble learning*.mp. | 3973 | Advanced | 0.77 |  |  |
| 107 | extreme learning machine*.mp. | 1622 | Advanced | 0.45 |  |  |
| 108 | federated learning*.mp. | 2105 | Advanced | 0.20 |  |  |
| 109 | feedforward neural network*.mp. | 1147 | Advanced | 0.27 |  |  |
| 110 | generative ai*.mp. | 2514 | Advanced | 2.96 |  |  |
| 111 | generative artificial intelligence*.mp. | 3289 | Advanced | 0.16 |  |  |
| 112 | graph neural network*.mp. | 4548 | Advanced | 0.36 |  |  |
| 113 | hierarchical learning.mp. | 150 | Advanced | 0.17 |  |  |
| 114 | hopfield neural network*.mp. | 315 | Advanced | 0.22 |  |  |
| 115 | image recognition.mp. | 2698 | Advanced | 0.52 |  |  |
| 116 | inductive machine learning.mp. | 23 | Advanced | 0.25 |  |  |
| 117 | intelligent machine?.mp. | 254 | Advanced | 0.12 |  |  |
| 118 | intelligent system*.mp. | 1871 | Advanced | 1.52 |  |  |
| 119 | machine assisted discovery.mp. | 2 | Advanced | 0.23 |  |  |
| 120 | (machine adj2 intelligence).mp. | 3382 | Advanced | 0.18 |  |  |
| 121 | machine learning algorithm*.mp. | 26372 | Advanced | 0.25 |  |  |
| 122 | machine learning.mp. | 189861 | Advanced | 0.26 |  |  |
| 123 | machine learnt.mp. | 14 | Advanced | 0.12 |  |  |
| 124 | machine prediction method*.mp. | 3 | Advanced | 0.53 |  |  |
| 125 | machine-assist* discover*.mp. | 2 | Advanced | 0.18 |  |  |
| 126 | multiple-instance learning algorithm*.mp. | 59 | Advanced | 0.39 |  |  |
| 127 | pattern analysis machine*.mp. | 153 | Advanced | 0.44 |  |  |
| 128 | predictive learning model*.mp. | 91 | Advanced | 0.46 |  |  |
| 129 | recurrent neural network*.mp. | 6217 | Advanced | 0.44 |  |  |
| 130 | reinforcement machine learning*.mp. | 78 | Advanced | 0.37 |  |  |
| 131 | representation machine learning*.mp. | 5 | Advanced | 0.19 |  |  |
| 132 | self-learning.mp. | 1726 | Advanced | 0.33 |  |  |
| 133 | (semisupervised adj2 (learn* or algorithm*)).mp. | 351 | Advanced | 0.44 |  |  |
| 134 | (semi-supervised adj2 (learn* or algorithm*)).mp. | 2167 | Advanced | 0.67 |  |  |
| 135 | supervised machine* learn*.mp. | 6500 | Advanced | 0.16 |  |  |
| 136 | support vector machine?.mp. | 38975 | Advanced | 0.32 |  |  |
| 137 | support vector network?.mp. | 7 | Advanced | 1.03 |  |  |
| 138 | (train adj2 (dataset or data-set)).mp. | 381 | Advanced | 0.74 |  |  |
| 139 | (train adj2 algorithm*).mp. | 516 | Advanced | 0.15 |  |  |
| 140 | transfer learning.mp. | 9557 | Advanced | 0.36 |  |  |
| 141 | transfer machine learning*.mp. | 18 | Advanced | 0.25 |  |  |
| 142 | (unsupervised adj2 (learn* or algorithm*)).mp. | 7939 | Advanced | 0.52 |  |  |
| 143 | (un-supervised adj2 (learn* or algorithm*)).mp. | 17 | Advanced | 0.51 |  |  |
| 144 | or/1-143 | 1603473 | Advanced | 0.82 |  |  |
| 145 | intersectional framework/ | 317 | Advanced | 0.05 |  |  |
| 146 | intersectional*.mp. | 7673 | Advanced | 0.66 |  |  |
| 147 | (intersecting adj5 identit*).mp. | 547 | Advanced | 0.76 |  |  |
| 148 | (interlocking adj3 system?).mp. | 224 | Advanced | 0.34 |  |  |
| 149 | (interlocking adj3 oppression?).mp. | 53 | Advanced | 0.40 |  |  |
| 150 | (overlapping adj5 identit*).mp. | 138 | Advanced | 0.21 |  |  |
| 151 | (overlapping adj2 social categor*).mp. | 3 | Advanced | 0.95 |  |  |
| 152 | (multiple adj2 social identit*).mp. | 123 | Advanced | 0.28 |  |  |
| 153 | (intersecting adj2 social categor*).mp. | 4 | Advanced | 0.20 |  |  |
| 154 | (multiple adj3 vulnerabilit*).mp. | 515 | Advanced | 0.52 |  |  |
| 155 | (intersecting adj3 vulnerabilit*).mp. | 73 | Advanced | 0.10 |  |  |
| 156 | (multiple adj3 intersection*).mp. | 458 | Advanced | 0.63 |  |  |
| 157 | (layered adj3 disadvantage?).mp. | 8 | Advanced | 0.59 |  |  |
| 158 | (complex adj2 social disadvantage?).mp. | 0 | Advanced | 0.36 |  |  |
| 159 | (Multidimensional adj3 inequalit*).mp. | 38 | Advanced | 0.48 |  |  |
| 160 | (Multi-dimensional adj3 inequalit*).mp. | 5 | Advanced | 0.27 |  |  |
| 161 | (Multifactorial adj3 disadvantage?).mp. | 4 | Advanced | 0.09 |  |  |
| 162 | (Multi-factorial adj3 disadvantage?).mp. | 0 | Advanced | 0.17 |  |  |
| 163 | (Compound adj3 disadvantage?).mp. | 40 | Advanced | 0.20 |  |  |
| 164 | (Cumulative adj3 disadvantage?).mp. | 383 | Advanced | 0.29 |  |  |
| 165 | (intersection between adj2 (race or racial or ethnic*)).mp. | 33 | Advanced | 1.93 |  |  |
| 166 | (social adj2 power).mp. | 1023 | Advanced | 0.39 |  |  |
| 167 | (interpersonal adj2 power).mp. | 78 | Advanced | 0.24 |  |  |
| 168 | (relational adj2 power).mp. | 71 | Advanced | 0.21 |  |  |
| 169 | (structural adj2 inequalit*).mp. | 886 | Advanced | 0.12 |  |  |
| 170 | (systemic adj2 (inequalit* or inequit*)).mp. | 817 | Advanced | 0.50 |  |  |
| 171 | (institutional adj2 inequalit*).mp. | 45 | Advanced | 0.17 |  |  |
| 172 | (embedded adj2 inequalit*).mp. | 26 | Advanced | 0.09 |  |  |
| 173 | or/145-172 | 12309 | Advanced | 0.02 |  |  |
| 174 | 144 and 173 | 1079 | Advanced | 0.09 |  |  |
| 175 | limit 174 to english language | 1063 | Advanced | 0.02 |  |  |

Embase <1974 to 2026 January 23>

| **#** | **Searches** | **Results** | **Type** | **Runtime** |  |  |
| --- | --- | --- | --- | --- | --- | --- |
|  | | | | | | |
| 1 | exp Artificial Intelligence/ | 181297 | Advanced | 2.34 |  |  |
| 2 | Big Data/ | 9257 | Advanced | 0.06 |  |  |
| 3 | medical technology/ | 39197 | Advanced | 0.00 |  |  |
| 4 | computer heuristics/ | 810 | Advanced | 0.01 |  |  |
| 5 | exp computer aided design/ | 74122 | Advanced | 0.54 |  |  |
| 6 | Data Aggregation/ | 1702 | Advanced | 0.03 |  |  |
| 7 | exp data analysis/ | 256740 | Advanced | 0.59 |  |  |
| 8 | exp data mining/ | 28626 | Advanced | 0.12 |  |  |
| 9 | Data Science/ | 2338 | Advanced | 0.00 |  |  |
| 10 | exp decision support system/ | 47231 | Advanced | 0.21 |  |  |
| 11 | "decision tree"/ | 32120 | Advanced | 0.03 |  |  |
| 12 | Deep Learning/ | 104472 | Advanced | 0.03 |  |  |
| 13 | exp Automation/ | 142282 | Advanced | 0.48 |  |  |
| 14 | expert system/ | 6171 | Advanced | 0.03 |  |  |
| 15 | exp image processing/ | 166630 | Advanced | 0.39 |  |  |
| 16 | exp machine learning/ | 695018 | Advanced | 4.53 |  |  |
| 17 | exp artificial neural network/ | 158765 | Advanced | 0.17 |  |  |
| 18 | mathematical computing/ | 54670 | Advanced | 0.04 |  |  |
| 19 | automated pattern recognition/ | 16708 | Advanced | 0.03 |  |  |
| 20 | exp signal processing/ | 296139 | Advanced | 0.96 |  |  |
| 21 | supervised machine learning/ | 7311 | Advanced | 0.03 |  |  |
| 22 | exp support vector machine/ | 62670 | Advanced | 0.03 |  |  |
| 23 | Unsupervised Machine Learning/ | 4840 | Advanced | 0.05 |  |  |
| 24 | "artif#cial intelligence".tw,kf. | 115884 | Advanced | 0.77 |  |  |
| 25 | "big data".tw,kf. | 21361 | Advanced | 1.12 |  |  |
| 26 | ("AI" and intelligence).tw. | 50886 | Advanced | 0.23 |  |  |
| 27 | ((neural* adj2 network*) and computer).tw,kf. | 14030 | Advanced | 1.75 |  |  |
| 28 | ((text? or textual*) adj2 analy*).tw,kf. | 7481 | Advanced | 3.48 |  |  |
| 29 | (algor#th* adj3 (comput* or machine? or intelligen* or art#ficial* or analy*)).tw,kf. | 84153 | Advanced | 4.12 |  |  |
| 30 | (art#ficial* adj2 intelligen*).tw,kf. | 116809 | Advanced | 0.08 |  |  |
| 31 | (automat* adj2 algor#th*).tw,kf. | 12924 | Advanced | 0.89 |  |  |
| 32 | (automat* adj2 pattern? adj2 analy*).tw,kf. | 84 | Advanced | 0.99 |  |  |
| 33 | (automat* adj2 pattern? adj2 recog*).tw,kf. | 246 | Advanced | 1.10 |  |  |
| 34 | (automat* adj2 pattern? adj2 classifi*).tw,kf. | 35 | Advanced | 0.67 |  |  |
| 35 | (automat*4 and (computer* or technolog*)).tw,kf. | 74788 | Advanced | 0.77 |  |  |
| 36 | (comput* adj1 analy*).tw,kf. | 28849 | Advanced | 0.69 |  |  |
| 37 | (comput* adj2 knowledge adj2 (acquisition or representation*)).tw,kf. | 23 | Advanced | 1.09 |  |  |
| 38 | (comput* adj2 pattern? adj2 analy*).tw,kf. | 101 | Advanced | 0.70 |  |  |
| 39 | (comput* adj2 pattern? adj2 recog*).tw,kf. | 220 | Advanced | 0.23 |  |  |
| 40 | (comput* adj2 pattern? adj2 classifi*).tw,kf. | 11 | Advanced | 0.23 |  |  |
| 41 | (comput* adj2 reasoning*).tw,kf. | 151 | Advanced | 0.25 |  |  |
| 42 | (computat* adj2 intelligen*).tw,kf. | 1165 | Advanced | 0.15 |  |  |
| 43 | (computational adj1 approach??).tw,kf. | 19948 | Advanced | 0.79 |  |  |
| 44 | (computational adj1 repurpos*).tw,kf. | 67 | Advanced | 0.23 |  |  |
| 45 | (computer* adj2 vision*).tw,kf. | 14271 | Advanced | 0.53 |  |  |
| 46 | (computer* adj2 heuristic*).tw,kf. | 16 | Advanced | 0.23 |  |  |
| 47 | (data adj1 mining).tw,kf. | 21688 | Advanced | 0.50 |  |  |
| 48 | (deep* adj2 learn*).tw,kf. | 124397 | Advanced | 1.37 |  |  |
| 49 | (deep* adj2 network*).tw,kf. | 25047 | Advanced | 0.13 |  |  |
| 50 | (digital adj2 technolog*).tw,kf. | 15841 | Advanced | 0.11 |  |  |
| 51 | (hierarchic* adj1 learn*).tw,kf. | 348 | Advanced | 0.34 |  |  |
| 52 | (inductive* adj1 machine* adj1 learn*).tw,kf. | 28 | Advanced | 0.36 |  |  |
| 53 | (information?? adj1 technolog*).tw,kf. | 25022 | Advanced | 0.60 |  |  |
| 54 | (knowledge acqui* and computer*).tw,kf. | 295 | Advanced | 0.74 |  |  |
| 55 | (knowledge discovery and computer).tw,kf. | 110 | Advanced | 0.30 |  |  |
| 56 | (knowledge represent* and computer).tw,kf. | 244 | Advanced | 0.74 |  |  |
| 57 | (learning adj1 model* adj1 algorithm*).tw,kf. | 30 | Advanced | 2.03 |  |  |
| 58 | (learning adj2 algorithm*).tw,kf. | 53914 | Advanced | 0.18 |  |  |
| 59 | (learning adj3 labeled data).tw,kf. | 121 | Advanced | 0.56 |  |  |
| 60 | ((logistic* or logit) adj2 regress*).tw,kf. | 778409 | Advanced | 1.23 |  |  |
| 61 | (machine? adj2 assist* adj2 discover*).tw,kf. | 33 | Advanced | 0.76 |  |  |
| 62 | (machine? adj2 intelligen*).tw,kf. | 4338 | Advanced | 0.05 |  |  |
| 63 | (machine? adj2 learn*).tw,kf. | 211963 | Advanced | 0.15 |  |  |
| 64 | (ML adj2 (framework? or tool*)).tw,kf. | 1606 | Advanced | 1.16 |  |  |
| 65 | ML algorithm*.tw,kf. | 7651 | Advanced | 0.15 |  |  |
| 66 | ML application*.tw,kf. | 733 | Advanced | 0.45 |  |  |
| 67 | ML method*.tw,kf. | 5639 | Advanced | 3.25 |  |  |
| 68 | MLP.tw,kf. | 7295 | Advanced | 0.12 |  |  |
| 69 | (native adj2 bayes).tw,kf. | 10 | Advanced | 0.47 |  |  |
| 70 | (learn* adj2 bayes).tw,kf. | 102 | Advanced | 0.10 |  |  |
| 71 | (neural* adj2 network* adj2 (model* or comput*)).tw,kf. | 21045 | Advanced | 1.15 |  |  |
| 72 | (predictive adj2 algor#th*).tw,kf. | 3678 | Advanced | 0.38 |  |  |
| 73 | (predictive adj2 analy*).tw,kf. | 14455 | Advanced | 0.61 |  |  |
| 74 | (random* adj2 forest?).tw,kf. | 54005 | Advanced | 1.74 |  |  |
| 75 | (regulari* adj3 algorithm*).tw,kf. | 870 | Advanced | 0.22 |  |  |
| 76 | (regress* adj2 tree?).tw,kf. | 9247 | Advanced | 0.56 |  |  |
| 77 | (ridge adj2 regress*).tw,kf. | 2675 | Advanced | 0.28 |  |  |
| 78 | RVM.tw,kf. | 1375 | Advanced | 0.16 |  |  |
| 79 | (structural equation* adj2 model*).tw,kf. | 41297 | Advanced | 1.15 |  |  |
| 80 | (support adj2 vector).tw,kf. | 47704 | Advanced | 0.74 |  |  |
| 81 | (vector? adj2 machine?).tw,kf. | 43995 | Advanced | 0.23 |  |  |
| 82 | (vector? adj2 network*).tw,kf. | 989 | Advanced | 0.10 |  |  |
| 83 | adaptive algorithm*.tw,kf. | 1040 | Advanced | 0.27 |  |  |
| 84 | art#ficial neural network*.tw,kf. | 26992 | Advanced | 0.29 |  |  |
| 85 | artificial intelligence*.tw,kf. | 116019 | Advanced | 0.18 |  |  |
| 86 | Bayesian network*.tw,kf. | 8408 | Advanced | 0.18 |  |  |
| 87 | big health* data.tw,kf. | 77 | Advanced | 1.87 |  |  |
| 88 | chatgpt*.tw,kf. | 9649 | Advanced | 0.07 |  |  |
| 89 | (classification? adj2 algorithm*).tw,kf. | 9255 | Advanced | 0.22 |  |  |
| 90 | clustering algorithm*.tw,kf. | 8483 | Advanced | 0.23 |  |  |
| 91 | compression algorithm*.tw,kf. | 798 | Advanced | 0.11 |  |  |
| 92 | computational intelligence.tw,kf. | 788 | Advanced | 0.11 |  |  |
| 93 | computer cellular neural network*.tw,kf. | 0 | Advanced | 0.24 |  |  |
| 94 | computer reasoning.tw,kf. | 15 | Advanced | 0.09 |  |  |
| 95 | computer vision system?.tw,kf. | 462 | Advanced | 0.90 |  |  |
| 96 | convolutional neural network*.tw,kf. | 47887 | Advanced | 0.23 |  |  |
| 97 | (crossvalidation or cross-validation).tw,kf. | 57176 | Advanced | 0.58 |  |  |
| 98 | data analytic?.tw,kf. | 5096 | Advanced | 0.38 |  |  |
| 99 | data driven science?.tw,kf. | 88 | Advanced | 1.09 |  |  |
| 100 | data-driven science?.tw,kf. | 88 | Advanced | 0.36 |  |  |
| 101 | deep learning.tw,kf. | 120165 | Advanced | 0.19 |  |  |
| 102 | detection algorithm*.tw,kf. | 9665 | Advanced | 0.53 |  |  |
| 103 | digital intervention?.tw,kf. | 3949 | Advanced | 0.55 |  |  |
| 104 | ensemble learning*.tw,kf. | 4357 | Advanced | 0.40 |  |  |
| 105 | extreme learning machine*.tw,kf. | 2113 | Advanced | 0.32 |  |  |
| 106 | federated learning*.tw,kf. | 2146 | Advanced | 0.23 |  |  |
| 107 | feedforward neural network*.tw,kf. | 1246 | Advanced | 0.25 |  |  |
| 108 | generative ai*.tw,kf. | 4121 | Advanced | 2.62 |  |  |
| 109 | generative artificial intelligence*.tw,kf. | 2125 | Advanced | 0.09 |  |  |
| 110 | graph neural network*.tw,kf. | 4914 | Advanced | 0.31 |  |  |
| 111 | hierarchical learning.tw,kf. | 181 | Advanced | 0.11 |  |  |
| 112 | hopfield neural network*.tw,kf. | 374 | Advanced | 0.25 |  |  |
| 113 | image recognition.tw,kf. | 3200 | Advanced | 0.31 |  |  |
| 114 | inductive machine learning.tw,kf. | 27 | Advanced | 0.16 |  |  |
| 115 | intelligent machine?.tw,kf. | 276 | Advanced | 0.06 |  |  |
| 116 | intelligent system*.tw,kf. | 2024 | Advanced | 2.02 |  |  |
| 117 | machine assisted discovery.tw,kf. | 2 | Advanced | 0.13 |  |  |
| 118 | (machine adj2 intelligence).tw,kf. | 3855 | Advanced | 0.09 |  |  |
| 119 | machine learning algorithm*.tw,kf. | 32144 | Advanced | 0.16 |  |  |
| 120 | machine learning.tw,kf. | 209068 | Advanced | 0.20 |  |  |
| 121 | machine learnt.tw,kf. | 16 | Advanced | 0.07 |  |  |
| 122 | machine prediction method*.tw,kf. | 4 | Advanced | 0.65 |  |  |
| 123 | machine-assist* discover*.tw,kf. | 2 | Advanced | 0.12 |  |  |
| 124 | multiple-instance learning algorithm*.tw,kf. | 22 | Advanced | 0.27 |  |  |
| 125 | pattern analysis machine*.tw,kf. | 5 | Advanced | 0.39 |  |  |
| 126 | predictive learning model*.tw,kf. | 43 | Advanced | 0.41 |  |  |
| 127 | recurrent neural network*.tw,kf. | 7017 | Advanced | 0.40 |  |  |
| 128 | reinforcement machine learning*.tw,kf. | 12 | Advanced | 0.23 |  |  |
| 129 | representation machine learning*.tw,kf. | 5 | Advanced | 0.12 |  |  |
| 130 | self-learning.tw,kf. | 2276 | Advanced | 0.19 |  |  |
| 131 | (semisupervised adj2 (learn* or algorithm*)).tw,kf. | 326 | Advanced | 0.35 |  |  |
| 132 | (semi-supervised adj2 (learn* or algorithm*)).tw,kf. | 2642 | Advanced | 0.61 |  |  |
| 133 | supervised machine* learn*.tw,kf. | 5322 | Advanced | 0.12 |  |  |
| 134 | support vector machine?.tw,kf. | 43457 | Advanced | 0.18 |  |  |
| 135 | support vector network?.tw,kf. | 10 | Advanced | 0.41 |  |  |
| 136 | (train adj2 (dataset or data-set)).tw,kf. | 494 | Advanced | 0.82 |  |  |
| 137 | (train adj2 algorithm*).tw,kf. | 802 | Advanced | 0.07 |  |  |
| 138 | transfer learning.tw,kf. | 10480 | Advanced | 0.46 |  |  |
| 139 | transfer machine learning*.tw,kf. | 15 | Advanced | 0.15 |  |  |
| 140 | (unsupervised adj2 (learn* or algorithm*)).tw,kf. | 9246 | Advanced | 0.46 |  |  |
| 141 | (un-supervised adj2 (learn* or algorithm*)).tw,kf. | 28 | Advanced | 0.38 |  |  |
| 142 | or/1-141 | 2679461 | Advanced | 0.75 |  |  |
| 143 | intersectionality/ | 2344 | Advanced | 0.06 |  |  |
| 144 | intersectional*.tw,kf. | 8193 | Advanced | 0.19 |  |  |
| 145 | (intersecting adj5 identit*).tw,kf. | 551 | Advanced | 0.36 |  |  |
| 146 | (interlocking adj3 system?).tw,kf. | 253 | Advanced | 0.31 |  |  |
| 147 | (interlocking adj3 oppression?).tw,kf. | 53 | Advanced | 0.20 |  |  |
| 148 | (overlapping adj5 identit*).tw,kf. | 147 | Advanced | 0.17 |  |  |
| 149 | (overlapping adj2 social categor*).tw,kf. | 2 | Advanced | 0.58 |  |  |
| 150 | (multiple adj2 social identit*).tw,kf. | 117 | Advanced | 0.22 |  |  |
| 151 | (intersecting adj2 social categor*).tw,kf. | 4 | Advanced | 0.17 |  |  |
| 152 | (multiple adj3 vulnerabilit*).tw,kf. | 663 | Advanced | 0.51 |  |  |
| 153 | (intersecting adj3 vulnerabilit*).tw,kf. | 83 | Advanced | 0.05 |  |  |
| 154 | (multiple adj3 intersection*).tw,kf. | 485 | Advanced | 0.20 |  |  |
| 155 | (layered adj3 disadvantage?).tw,kf. | 10 | Advanced | 0.39 |  |  |
| 156 | (complex adj2 social disadvantage?).tw,kf. | 0 | Advanced | 0.30 |  |  |
| 157 | (Multidimensional adj3 inequalit*).tw,kf. | 35 | Advanced | 0.38 |  |  |
| 158 | (Multi-dimensional adj3 inequalit*).tw,kf. | 4 | Advanced | 0.34 |  |  |
| 159 | (Multifactorial adj3 disadvantage?).tw,kf. | 4 | Advanced | 0.12 |  |  |
| 160 | (Multi-factorial adj3 disadvantage?).tw,kf. | 0 | Advanced | 0.14 |  |  |
| 161 | (Compound adj3 disadvantage?).tw,kf. | 44 | Advanced | 0.17 |  |  |
| 162 | (Cumulative adj3 disadvantage?).tw,kf. | 398 | Advanced | 0.17 |  |  |
| 163 | (intersection between adj2 (race or racial or ethnic*)).tw,kf. | 49 | Advanced | 1.99 |  |  |
| 164 | (social adj2 power).tw,kf. | 1060 | Advanced | 0.33 |  |  |
| 165 | (interpersonal adj2 power).tw,kf. | 80 | Advanced | 0.16 |  |  |
| 166 | (relational adj2 power).tw,kf. | 72 | Advanced | 0.09 |  |  |
| 167 | (structural adj2 inequalit*).tw,kf. | 898 | Advanced | 0.08 |  |  |
| 168 | (systemic adj2 (inequalit* or inequit*)).tw,kf. | 969 | Advanced | 0.18 |  |  |
| 169 | (institutional adj2 inequalit*).tw,kf. | 39 | Advanced | 0.16 |  |  |
| 170 | (embedded adj2 inequalit*).tw,kf. | 25 | Advanced | 0.05 |  |  |
| 171 | or/143-170 | 13680 | Advanced | 0.02 |  |  |
| 172 | 142 and 171 | 1533 | Advanced | 0.13 |  |  |
| 173 | limit 172 to english language | 1510 | Advanced | 0.06 |  |  |
| 174 | limit 173 to (conference abstract or conference paper or "conference review" or conference proceeding) | 248 | Advanced | 0.92 |  |  |
| 175 | 173 not 174 | 1262 | Advanced | 0.00 |  |  |
| 176 | remove duplicates from 175 | 1254 | Advanced | 5.65 |  |  |

Ovid Emcare Nursing <1995 to Present>

| **#** | **Searches** | **Results** | **Type** | **Runtime** |  |  |
| --- | --- | --- | --- | --- | --- | --- |
|  | | | | | | |
| 1 | exp Artificial Intelligence/ | 56574 | Advanced | 1.32 |  |  |
| 2 | Big Data/ | 3518 | Advanced | 0.03 |  |  |
| 3 | medical technology/ | 14801 | Advanced | 0.02 |  |  |
| 4 | computer heuristics/ | 359 | Advanced | 0.01 |  |  |
| 5 | exp computer aided design/ | 16801 | Advanced | 0.39 |  |  |
| 6 | Data Aggregation/ | 512 | Advanced | 0.02 |  |  |
| 7 | exp data analysis/ | 103299 | Advanced | 0.27 |  |  |
| 8 | exp data mining/ | 7730 | Advanced | 0.03 |  |  |
| 9 | Data Science/ | 933 | Advanced | 0.00 |  |  |
| 10 | exp decision support system/ | 20553 | Advanced | 0.08 |  |  |
| 11 | "decision tree"/ | 10355 | Advanced | 0.01 |  |  |
| 12 | Deep Learning/ | 34457 | Advanced | 0.01 |  |  |
| 13 | exp Automation/ | 38897 | Advanced | 0.35 |  |  |
| 14 | expert system/ | 2222 | Advanced | 0.01 |  |  |
| 15 | exp image processing/ | 49184 | Advanced | 0.20 |  |  |
| 16 | exp machine learning/ | 178901 | Advanced | 2.27 |  |  |
| 17 | exp artificial neural network/ | 46117 | Advanced | 0.04 |  |  |
| 18 | mathematical computing/ | 13396 | Advanced | 0.01 |  |  |
| 19 | automated pattern recognition/ | 421 | Advanced | 0.01 |  |  |
| 20 | exp signal processing/ | 83705 | Advanced | 0.39 |  |  |
| 21 | supervised machine learning/ | 2210 | Advanced | 0.01 |  |  |
| 22 | exp support vector machine/ | 21946 | Advanced | 0.02 |  |  |
| 23 | Unsupervised Machine Learning/ | 1267 | Advanced | 0.01 |  |  |
| 24 | "artif#cial intelligence".tw,kf. | 36242 | Advanced | 0.32 |  |  |
| 25 | "big data".tw,kf. | 6750 | Advanced | 0.31 |  |  |
| 26 | ("AI" and intelligence).tw. | 15048 | Advanced | 0.10 |  |  |
| 27 | ((neural* adj2 network*) and computer).tw,kf. | 3940 | Advanced | 0.52 |  |  |
| 28 | ((text? or textual*) adj2 analy*).tw,kf. | 4586 | Advanced | 1.18 |  |  |
| 29 | (algor#th* adj3 (comput* or machine? or intelligen* or art#ficial* or analy*)).tw,kf. | 21801 | Advanced | 1.23 |  |  |
| 30 | (art#ficial* adj2 intelligen*).tw,kf. | 36457 | Advanced | 0.02 |  |  |
| 31 | (automat* adj2 algor#th*).tw,kf. | 3357 | Advanced | 0.22 |  |  |
| 32 | (automat* adj2 pattern? adj2 analy*).tw,kf. | 15 | Advanced | 0.35 |  |  |
| 33 | (automat* adj2 pattern? adj2 recog*).tw,kf. | 92 | Advanced | 0.28 |  |  |
| 34 | (automat* adj2 pattern? adj2 classifi*).tw,kf. | 11 | Advanced | 0.29 |  |  |
| 35 | (automat*4 and (computer* or technolog*)).tw,kf. | 19224 | Advanced | 0.46 |  |  |
| 36 | (comput* adj1 analy*).tw,kf. | 4203 | Advanced | 0.15 |  |  |
| 37 | (comput* adj2 knowledge adj2 (acquisition or representation*)).tw,kf. | 22 | Advanced | 0.39 |  |  |
| 38 | (comput* adj2 pattern? adj2 analy*).tw,kf. | 17 | Advanced | 0.18 |  |  |
| 39 | (comput* adj2 pattern? adj2 recog*).tw,kf. | 27 | Advanced | 0.05 |  |  |
| 40 | (comput* adj2 pattern? adj2 classifi*).tw,kf. | 0 | Advanced | 0.06 |  |  |
| 41 | (comput* adj2 reasoning*).tw,kf. | 56 | Advanced | 0.11 |  |  |
| 42 | (computat* adj2 intelligen*).tw,kf. | 287 | Advanced | 0.04 |  |  |
| 43 | (computational adj1 approach??).tw,kf. | 2991 | Advanced | 0.30 |  |  |
| 44 | (computational adj1 repurpos*).tw,kf. | 13 | Advanced | 0.05 |  |  |
| 45 | (computer* adj2 vision*).tw,kf. | 3266 | Advanced | 0.15 |  |  |
| 46 | (computer* adj2 heuristic*).tw,kf. | 7 | Advanced | 0.11 |  |  |
| 47 | (data adj1 mining).tw,kf. | 6288 | Advanced | 0.17 |  |  |
| 48 | (deep* adj2 learn*).tw,kf. | 36563 | Advanced | 0.49 |  |  |
| 49 | (deep* adj2 network*).tw,kf. | 6561 | Advanced | 0.03 |  |  |
| 50 | (digital adj2 technolog*).tw,kf. | 8025 | Advanced | 0.13 |  |  |
| 51 | (hierarchic* adj1 learn*).tw,kf. | 82 | Advanced | 0.11 |  |  |
| 52 | (inductive* adj1 machine* adj1 learn*).tw,kf. | 12 | Advanced | 0.14 |  |  |
| 53 | (information?? adj1 technolog*).tw,kf. | 14415 | Advanced | 0.27 |  |  |
| 54 | (knowledge acqui* and computer*).tw,kf. | 156 | Advanced | 0.17 |  |  |
| 55 | (knowledge discovery and computer).tw,kf. | 42 | Advanced | 0.11 |  |  |
| 56 | (knowledge represent* and computer).tw,kf. | 156 | Advanced | 0.17 |  |  |
| 57 | (learning adj1 model* adj1 algorithm*).tw,kf. | 5 | Advanced | 0.74 |  |  |
| 58 | (learning adj2 algorithm*).tw,kf. | 14781 | Advanced | 0.04 |  |  |
| 59 | (learning adj3 labeled data).tw,kf. | 37 | Advanced | 0.13 |  |  |
| 60 | ((logistic* or logit) adj2 regress*).tw,kf. | 269082 | Advanced | 0.42 |  |  |
| 61 | (machine? adj2 assist* adj2 discover*).tw,kf. | 2 | Advanced | 0.36 |  |  |
| 62 | (machine? adj2 intelligen*).tw,kf. | 1112 | Advanced | 0.02 |  |  |
| 63 | (machine? adj2 learn*).tw,kf. | 59213 | Advanced | 0.04 |  |  |
| 64 | (ML adj2 (framework? or tool*)).tw,kf. | 349 | Advanced | 0.52 |  |  |
| 65 | ML algorithm*.tw,kf. | 2198 | Advanced | 0.03 |  |  |
| 66 | ML application*.tw,kf. | 177 | Advanced | 0.20 |  |  |
| 67 | ML method*.tw,kf. | 1247 | Advanced | 0.83 |  |  |
| 68 | MLP.tw,kf. | 1835 | Advanced | 0.03 |  |  |
| 69 | (native adj2 bayes).tw,kf. | 2 | Advanced | 0.12 |  |  |
| 70 | (learn* adj2 bayes).tw,kf. | 28 | Advanced | 0.03 |  |  |
| 71 | (neural* adj2 network* adj2 (model* or comput*)).tw,kf. | 5054 | Advanced | 0.29 |  |  |
| 72 | (predictive adj2 algor#th*).tw,kf. | 841 | Advanced | 0.12 |  |  |
| 73 | (predictive adj2 analy*).tw,kf. | 3889 | Advanced | 0.14 |  |  |
| 74 | (random* adj2 forest?).tw,kf. | 15797 | Advanced | 0.46 |  |  |
| 75 | (regulari* adj3 algorithm*).tw,kf. | 219 | Advanced | 0.03 |  |  |
| 76 | (regress* adj2 tree?).tw,kf. | 2846 | Advanced | 0.13 |  |  |
| 77 | (ridge adj2 regress*).tw,kf. | 702 | Advanced | 0.09 |  |  |
| 78 | RVM.tw,kf. | 269 | Advanced | 0.05 |  |  |
| 79 | (structural equation* adj2 model*).tw,kf. | 29930 | Advanced | 0.32 |  |  |
| 80 | (support adj2 vector).tw,kf. | 14771 | Advanced | 0.20 |  |  |
| 81 | (vector? adj2 machine?).tw,kf. | 13749 | Advanced | 0.05 |  |  |
| 82 | (vector? adj2 network*).tw,kf. | 297 | Advanced | 0.02 |  |  |
| 83 | adaptive algorithm*.tw,kf. | 288 | Advanced | 0.09 |  |  |
| 84 | art#ficial neural network*.tw,kf. | 6648 | Advanced | 0.06 |  |  |
| 85 | artificial intelligence*.tw,kf. | 36269 | Advanced | 0.04 |  |  |
| 86 | Bayesian network*.tw,kf. | 2796 | Advanced | 0.04 |  |  |
| 87 | big health* data.tw,kf. | 42 | Advanced | 1.08 |  |  |
| 88 | chatgpt*.tw,kf. | 3734 | Advanced | 0.07 |  |  |
| 89 | (classification? adj2 algorithm*).tw,kf. | 2704 | Advanced | 0.07 |  |  |
| 90 | clustering algorithm*.tw,kf. | 2236 | Advanced | 0.07 |  |  |
| 91 | compression algorithm*.tw,kf. | 287 | Advanced | 0.07 |  |  |
| 92 | computational intelligence.tw,kf. | 202 | Advanced | 0.02 |  |  |
| 93 | computer cellular neural network*.tw,kf. | 0 | Advanced | 0.12 |  |  |
| 94 | computer reasoning.tw,kf. | 6 | Advanced | 0.01 |  |  |
| 95 | computer vision system?.tw,kf. | 106 | Advanced | 0.37 |  |  |
| 96 | convolutional neural network*.tw,kf. | 13867 | Advanced | 0.10 |  |  |
| 97 | (crossvalidation or cross-validation).tw,kf. | 15415 | Advanced | 0.22 |  |  |
| 98 | data analytic?.tw,kf. | 1857 | Advanced | 0.09 |  |  |
| 99 | data driven science?.tw,kf. | 28 | Advanced | 0.32 |  |  |
| 100 | data-driven science?.tw,kf. | 28 | Advanced | 0.08 |  |  |
| 101 | deep learning.tw,kf. | 35410 | Advanced | 0.04 |  |  |
| 102 | detection algorithm*.tw,kf. | 2595 | Advanced | 0.11 |  |  |
| 103 | digital intervention?.tw,kf. | 1919 | Advanced | 0.36 |  |  |
| 104 | ensemble learning*.tw,kf. | 1582 | Advanced | 0.12 |  |  |
| 105 | extreme learning machine*.tw,kf. | 477 | Advanced | 0.09 |  |  |
| 106 | federated learning*.tw,kf. | 719 | Advanced | 0.06 |  |  |
| 107 | feedforward neural network*.tw,kf. | 257 | Advanced | 0.05 |  |  |
| 108 | generative ai*.tw,kf. | 1174 | Advanced | 0.65 |  |  |
| 109 | generative artificial intelligence*.tw,kf. | 999 | Advanced | 0.02 |  |  |
| 110 | graph neural network*.tw,kf. | 1236 | Advanced | 0.09 |  |  |
| 111 | hierarchical learning.tw,kf. | 47 | Advanced | 0.04 |  |  |
| 112 | hopfield neural network*.tw,kf. | 23 | Advanced | 0.05 |  |  |
| 113 | image recognition.tw,kf. | 689 | Advanced | 0.09 |  |  |
| 114 | inductive machine learning.tw,kf. | 11 | Advanced | 0.03 |  |  |
| 115 | intelligent machine?.tw,kf. | 71 | Advanced | 0.01 |  |  |
| 116 | intelligent system*.tw,kf. | 614 | Advanced | 0.39 |  |  |
| 117 | machine assisted discovery.tw,kf. | 1 | Advanced | 0.03 |  |  |
| 118 | (machine adj2 intelligence).tw,kf. | 992 | Advanced | 0.02 |  |  |
| 119 | machine learning algorithm*.tw,kf. | 9464 | Advanced | 0.04 |  |  |
| 120 | machine learning.tw,kf. | 58658 | Advanced | 0.04 |  |  |
| 121 | machine learnt.tw,kf. | 3 | Advanced | 0.01 |  |  |
| 122 | machine prediction method*.tw,kf. | 0 | Advanced | 0.15 |  |  |
| 123 | machine-assist* discover*.tw,kf. | 1 | Advanced | 0.03 |  |  |
| 124 | multiple-instance learning algorithm*.tw,kf. | 7 | Advanced | 0.15 |  |  |
| 125 | pattern analysis machine*.tw,kf. | 0 | Advanced | 0.09 |  |  |
| 126 | predictive learning model*.tw,kf. | 12 | Advanced | 0.10 |  |  |
| 127 | recurrent neural network*.tw,kf. | 1568 | Advanced | 0.07 |  |  |
| 128 | reinforcement machine learning*.tw,kf. | 1 | Advanced | 0.06 |  |  |
| 129 | representation machine learning*.tw,kf. | 1 | Advanced | 0.04 |  |  |
| 130 | self-learning.tw,kf. | 839 | Advanced | 0.15 |  |  |
| 131 | (semisupervised adj2 (learn* or algorithm*)).tw,kf. | 52 | Advanced | 0.11 |  |  |
| 132 | (semi-supervised adj2 (learn* or algorithm*)).tw,kf. | 750 | Advanced | 0.17 |  |  |
| 133 | supervised machine* learn*.tw,kf. | 1608 | Advanced | 0.03 |  |  |
| 134 | support vector machine?.tw,kf. | 13613 | Advanced | 0.04 |  |  |
| 135 | support vector network?.tw,kf. | 3 | Advanced | 0.12 |  |  |
| 136 | (train adj2 (dataset or data-set)).tw,kf. | 109 | Advanced | 0.22 |  |  |
| 137 | (train adj2 algorithm*).tw,kf. | 193 | Advanced | 0.02 |  |  |
| 138 | transfer learning.tw,kf. | 3322 | Advanced | 0.10 |  |  |
| 139 | transfer machine learning*.tw,kf. | 6 | Advanced | 0.04 |  |  |
| 140 | (unsupervised adj2 (learn* or algorithm*)).tw,kf. | 2160 | Advanced | 0.16 |  |  |
| 141 | (un-supervised adj2 (learn* or algorithm*)).tw,kf. | 9 | Advanced | 0.16 |  |  |
| 142 | or/1-141 | 822173 | Advanced | 0.24 |  |  |
| 143 | intersectionality/ | 2905 | Advanced | 0.02 |  |  |
| 144 | intersectional*.tw,kf. | 7038 | Advanced | 0.05 |  |  |
| 145 | (intersecting adj5 identit*).tw,kf. | 550 | Advanced | 0.11 |  |  |
| 146 | (interlocking adj3 system?).tw,kf. | 117 | Advanced | 0.08 |  |  |
| 147 | (interlocking adj3 oppression?).tw,kf. | 60 | Advanced | 0.08 |  |  |
| 148 | (overlapping adj5 identit*).tw,kf. | 68 | Advanced | 0.07 |  |  |
| 149 | (overlapping adj2 social categor*).tw,kf. | 2 | Advanced | 0.39 |  |  |
| 150 | (multiple adj2 social identit*).tw,kf. | 114 | Advanced | 0.08 |  |  |
| 151 | (intersecting adj2 social categor*).tw,kf. | 10 | Advanced | 0.05 |  |  |
| 152 | (multiple adj3 vulnerabilit*).tw,kf. | 279 | Advanced | 0.14 |  |  |
| 153 | (intersecting adj3 vulnerabilit*).tw,kf. | 45 | Advanced | 0.01 |  |  |
| 154 | (multiple adj3 intersection*).tw,kf. | 267 | Advanced | 0.05 |  |  |
| 155 | (layered adj3 disadvantage?).tw,kf. | 3 | Advanced | 0.12 |  |  |
| 156 | (complex adj2 social disadvantage?).tw,kf. | 0 | Advanced | 0.15 |  |  |
| 157 | (Multidimensional adj3 inequalit*).tw,kf. | 17 | Advanced | 0.13 |  |  |
| 158 | (Multi-dimensional adj3 inequalit*).tw,kf. | 4 | Advanced | 0.10 |  |  |
| 159 | (Multifactorial adj3 disadvantage?).tw,kf. | 3 | Advanced | 0.03 |  |  |
| 160 | (Multi-factorial adj3 disadvantage?).tw,kf. | 0 | Advanced | 0.07 |  |  |
| 161 | (Compound adj3 disadvantage?).tw,kf. | 19 | Advanced | 0.03 |  |  |
| 162 | (Cumulative adj3 disadvantage?).tw,kf. | 297 | Advanced | 0.05 |  |  |
| 163 | (intersection between adj2 (race or racial or ethnic*)).tw,kf. | 30 | Advanced | 0.54 |  |  |
| 164 | (social adj2 power).tw,kf. | 909 | Advanced | 0.14 |  |  |
| 165 | (interpersonal adj2 power).tw,kf. | 106 | Advanced | 0.08 |  |  |
| 166 | (relational adj2 power).tw,kf. | 95 | Advanced | 0.05 |  |  |
| 167 | (structural adj2 inequalit*).tw,kf. | 780 | Advanced | 0.03 |  |  |
| 168 | (systemic adj2 (inequalit* or inequit*)).tw,kf. | 593 | Advanced | 0.07 |  |  |
| 169 | (institutional adj2 inequalit*).tw,kf. | 40 | Advanced | 0.05 |  |  |
| 170 | (embedded adj2 inequalit*).tw,kf. | 32 | Advanced | 0.07 |  |  |
| 171 | or/143-170 | 10824 | Advanced | 0.01 |  |  |
| 172 | 142 and 171 | 1018 | Advanced | 0.04 |  |  |
| 173 | limit 172 to english language | 1002 | Advanced | 0.04 |  |  |
| 174 | limit 173 to (conference abstract or conference paper or "conference review" or conference proceeding) | 6 | Advanced | 0.28 |  |  |
| 175 | 173 not 174 | 996 | Advanced | 0.00 |  |  |
| 176 | remove duplicates from 175 | 993 | Advanced | 4.87 |  |  |

APA PsycInfo <1806 to January 2026 Week 4>

| **#** | **Searches** | **Results** | **Type** | **Runtime** |  |  |
| --- | --- | --- | --- | --- | --- | --- |
|  | | | | | | |
| 1 | exp artificial intelligence/ | 85507 | Advanced | 1.63 |  |  |
| 2 | big data/ | 2178 | Advanced | 0.01 |  |  |
| 3 | computer assisted design/ | 619 | Advanced | 0.03 |  |  |
| 4 | data analysis/ | 237 | Advanced | 0.00 |  |  |
| 5 | exp data mining/ | 5647 | Advanced | 0.06 |  |  |
| 6 | "decision tree algorithms"/ | 1072 | Advanced | 0.00 |  |  |
| 7 | exp deep neural networks/ | 4690 | Advanced | 0.03 |  |  |
| 8 | automation/ | 4029 | Advanced | 0.00 |  |  |
| 9 | exp expert systems/ | 10044 | Advanced | 0.03 |  |  |
| 10 | exp machine learning/ | 36975 | Advanced | 0.08 |  |  |
| 11 | support vector machine algorithms/ | 1232 | Advanced | 0.01 |  |  |
| 12 | "artif#cial intelligence".mp. | 23188 | Advanced | 0.55 |  |  |
| 13 | "big data".mp. | 4187 | Advanced | 0.45 |  |  |
| 14 | ("AI" and intelligence).tw. | 7072 | Advanced | 0.11 |  |  |
| 15 | ((neural* adj2 network*) and computer).mp. | 10069 | Advanced | 1.13 |  |  |
| 16 | ((text? or textual*) adj2 analy*).mp. | 7366 | Advanced | 1.64 |  |  |
| 17 | (algor#th* adj3 (comput* or machine? or intelligen* or art#ficial* or analy*)).mp. | 8342 | Advanced | 1.90 |  |  |
| 18 | (art#ficial* adj2 intelligen*).mp. | 23312 | Advanced | 0.06 |  |  |
| 19 | (automat* adj2 algor#th*).mp. | 756 | Advanced | 0.36 |  |  |
| 20 | (automat* adj2 pattern? adj2 analy*).mp. | 6 | Advanced | 0.56 |  |  |
| 21 | (automat* adj2 pattern? adj2 recog*).mp. | 1144 | Advanced | 0.60 |  |  |
| 22 | (automat* adj2 pattern? adj2 classifi*).mp. | 7 | Advanced | 0.62 |  |  |
| 23 | (automat*4 and (computer* or technolog*)).mp. | 16368 | Advanced | 1.05 |  |  |
| 24 | (comput* adj1 analy*).mp. | 2082 | Advanced | 0.14 |  |  |
| 25 | (comput* adj2 knowledge adj2 (acquisition or representation*)).mp. | 29 | Advanced | 0.78 |  |  |
| 26 | (comput* adj2 pattern? adj2 analy*).mp. | 21 | Advanced | 0.15 |  |  |
| 27 | (comput* adj2 pattern? adj2 recog*).mp. | 990 | Advanced | 0.11 |  |  |
| 28 | (comput* adj2 pattern? adj2 classifi*).mp. | 5 | Advanced | 0.09 |  |  |
| 29 | (comput* adj2 reasoning*).mp. | 156 | Advanced | 0.30 |  |  |
| 30 | (computat* adj2 intelligen*).mp. | 540 | Advanced | 0.17 |  |  |
| 31 | (computational adj1 approach??).mp. | 1407 | Advanced | 0.43 |  |  |
| 32 | (computational adj1 repurpos*).mp. | 0 | Advanced | 0.14 |  |  |
| 33 | (computer* adj2 vision*).mp. | 1909 | Advanced | 0.37 |  |  |
| 34 | (computer* adj2 heuristic*).mp. | 27 | Advanced | 0.34 |  |  |
| 35 | (data adj1 mining).mp. | 5181 | Advanced | 0.33 |  |  |
| 36 | (deep* adj2 learn*).mp. | 7198 | Advanced | 1.17 |  |  |
| 37 | (deep* adj2 network*).mp. | 3971 | Advanced | 0.06 |  |  |
| 38 | (digital adj2 technolog*).mp. | 10040 | Advanced | 0.31 |  |  |
| 39 | (hierarchic* adj1 learn*).mp. | 173 | Advanced | 0.32 |  |  |
| 40 | (inductive* adj1 machine* adj1 learn*).mp. | 14 | Advanced | 0.36 |  |  |
| 41 | (information?? adj1 technolog*).mp. | 12127 | Advanced | 0.41 |  |  |
| 42 | (knowledge acqui* and computer*).mp. | 665 | Advanced | 0.47 |  |  |
| 43 | (knowledge discovery and computer).mp. | 95 | Advanced | 0.31 |  |  |
| 44 | (knowledge represent* and computer).mp. | 426 | Advanced | 0.49 |  |  |
| 45 | (learning adj1 model* adj1 algorithm*).mp. | 8 | Advanced | 1.27 |  |  |
| 46 | (learning adj2 algorithm*).mp. | 5944 | Advanced | 0.14 |  |  |
| 47 | (learning adj3 labeled data).mp. | 20 | Advanced | 0.24 |  |  |
| 48 | ((logistic* or logit) adj2 regress*).mp. | 89039 | Advanced | 0.74 |  |  |
| 49 | (machine? adj2 assist* adj2 discover*).mp. | 1 | Advanced | 0.55 |  |  |
| 50 | (machine? adj2 intelligen*).mp. | 710 | Advanced | 0.06 |  |  |
| 51 | (machine? adj2 learn*).mp. | 24790 | Advanced | 0.13 |  |  |
| 52 | (ML adj2 (framework? or tool*)).mp. | 86 | Advanced | 1.01 |  |  |
| 53 | ML algorithm*.mp. | 342 | Advanced | 0.05 |  |  |
| 54 | ML application*.mp. | 44 | Advanced | 0.32 |  |  |
| 55 | ML method*.mp. | 259 | Advanced | 0.90 |  |  |
| 56 | MLP.mp. | 416 | Advanced | 0.06 |  |  |
| 57 | (native adj2 bayes).mp. | 0 | Advanced | 0.28 |  |  |
| 58 | (learn* adj2 bayes).mp. | 32 | Advanced | 0.12 |  |  |
| 59 | (neural* adj2 network* adj2 (model* or comput*)).mp. | 7410 | Advanced | 0.40 |  |  |
| 60 | (predictive adj2 algor#th*).mp. | 222 | Advanced | 0.23 |  |  |
| 61 | (predictive adj2 analy*).mp. | 1883 | Advanced | 0.18 |  |  |
| 62 | (random* adj2 forest?).mp. | 2744 | Advanced | 0.97 |  |  |
| 63 | (regulari* adj3 algorithm*).mp. | 107 | Advanced | 0.18 |  |  |
| 64 | (regress* adj2 tree?).mp. | 903 | Advanced | 0.29 |  |  |
| 65 | (ridge adj2 regress*).mp. | 280 | Advanced | 0.16 |  |  |
| 66 | RVM.mp. | 328 | Advanced | 0.11 |  |  |
| 67 | (structural equation* adj2 model*).mp. | 54945 | Advanced | 0.52 |  |  |
| 68 | (support adj2 vector).mp. | 4892 | Advanced | 0.47 |  |  |
| 69 | (vector? adj2 machine?).mp. | 4490 | Advanced | 0.20 |  |  |
| 70 | (vector? adj2 network*).mp. | 85 | Advanced | 0.05 |  |  |
| 71 | adaptive algorithm*.mp. | 147 | Advanced | 0.15 |  |  |
| 72 | art#ficial neural network*.mp. | 6893 | Advanced | 0.26 |  |  |
| 73 | artificial intelligence*.mp. | 23205 | Advanced | 0.37 |  |  |
| 74 | Bayesian network*.mp. | 1183 | Advanced | 0.14 |  |  |
| 75 | big health* data.mp. | 10 | Advanced | 1.24 |  |  |
| 76 | chatgpt*.mp. | 1058 | Advanced | 0.12 |  |  |
| 77 | (classification? adj2 algorithm*).mp. | 1100 | Advanced | 0.18 |  |  |
| 78 | clustering algorithm*.mp. | 1078 | Advanced | 0.16 |  |  |
| 79 | compression algorithm*.mp. | 50 | Advanced | 0.13 |  |  |
| 80 | computational intelligence.mp. | 423 | Advanced | 0.13 |  |  |
| 81 | computer cellular neural network*.mp. | 0 | Advanced | 0.30 |  |  |
| 82 | computer reasoning.mp. | 4 | Advanced | 0.12 |  |  |
| 83 | computer vision system?.mp. | 83 | Advanced | 0.68 |  |  |
| 84 | convolutional neural network*.mp. | 2494 | Advanced | 0.15 |  |  |
| 85 | (crossvalidation or cross-validation).mp. | 6119 | Advanced | 0.52 |  |  |
| 86 | data analytic?.mp. | 1848 | Advanced | 0.21 |  |  |
| 87 | data driven science?.mp. | 10 | Advanced | 0.95 |  |  |
| 88 | data-driven science?.mp. | 10 | Advanced | 0.14 |  |  |
| 89 | deep learning.mp. | 5961 | Advanced | 0.17 |  |  |
| 90 | detection algorithm*.mp. | 703 | Advanced | 0.22 |  |  |
| 91 | digital intervention?.mp. | 4095 | Advanced | 0.49 |  |  |
| 92 | ensemble learning*.mp. | 383 | Advanced | 0.38 |  |  |
| 93 | extreme learning machine*.mp. | 541 | Advanced | 0.25 |  |  |
| 94 | federated learning*.mp. | 89 | Advanced | 0.11 |  |  |
| 95 | feedforward neural network*.mp. | 288 | Advanced | 0.12 |  |  |
| 96 | generative ai*.mp. | 787 | Advanced | 1.00 |  |  |
| 97 | generative artificial intelligence*.mp. | 984 | Advanced | 0.13 |  |  |
| 98 | graph neural network*.mp. | 213 | Advanced | 0.23 |  |  |
| 99 | hierarchical learning.mp. | 98 | Advanced | 0.14 |  |  |
| 100 | hopfield neural network*.mp. | 127 | Advanced | 0.12 |  |  |
| 101 | image recognition.mp. | 329 | Advanced | 0.27 |  |  |
| 102 | inductive machine learning.mp. | 13 | Advanced | 0.20 |  |  |
| 103 | intelligent machine?.mp. | 214 | Advanced | 0.07 |  |  |
| 104 | intelligent system*.mp. | 834 | Advanced | 0.46 |  |  |
| 105 | machine assisted discovery.mp. | 0 | Advanced | 0.17 |  |  |
| 106 | (machine adj2 intelligence).mp. | 430 | Advanced | 0.18 |  |  |
| 107 | machine learning algorithm*.mp. | 2912 | Advanced | 0.22 |  |  |
| 108 | machine learning.mp. | 24524 | Advanced | 0.24 |  |  |
| 109 | machine learnt.mp. | 1 | Advanced | 0.06 |  |  |
| 110 | machine prediction method*.mp. | 0 | Advanced | 0.28 |  |  |
| 111 | machine-assist* discover*.mp. | 0 | Advanced | 0.08 |  |  |
| 112 | multiple-instance learning algorithm*.mp. | 2 | Advanced | 0.29 |  |  |
| 113 | pattern analysis machine*.mp. | 1 | Advanced | 0.22 |  |  |
| 114 | predictive learning model*.mp. | 5 | Advanced | 0.23 |  |  |
| 115 | recurrent neural network*.mp. | 1345 | Advanced | 0.21 |  |  |
| 116 | reinforcement machine learning*.mp. | 3 | Advanced | 0.32 |  |  |
| 117 | representation machine learning*.mp. | 2 | Advanced | 0.17 |  |  |
| 118 | self-learning.mp. | 588 | Advanced | 0.67 |  |  |
| 119 | (semisupervised adj2 (learn* or algorithm*)).mp. | 54 | Advanced | 0.24 |  |  |
| 120 | (semi-supervised adj2 (learn* or algorithm*)).mp. | 442 | Advanced | 0.39 |  |  |
| 121 | supervised machine* learn*.mp. | 607 | Advanced | 0.14 |  |  |
| 122 | support vector machine?.mp. | 4421 | Advanced | 0.12 |  |  |
| 123 | support vector network?.mp. | 2 | Advanced | 0.33 |  |  |
| 124 | (train adj2 (dataset or data-set)).mp. | 26 | Advanced | 0.39 |  |  |
| 125 | (train adj2 algorithm*).mp. | 67 | Advanced | 0.06 |  |  |
| 126 | transfer learning.mp. | 7494 | Advanced | 0.27 |  |  |
| 127 | transfer machine learning*.mp. | 1 | Advanced | 0.16 |  |  |
| 128 | (unsupervised adj2 (learn* or algorithm*)).mp. | 1349 | Advanced | 0.36 |  |  |
| 129 | (un-supervised adj2 (learn* or algorithm*)).mp. | 4 | Advanced | 0.37 |  |  |
| 130 | or/1-129 | 313760 | Advanced | 0.17 |  |  |
| 131 | intersectionality/ | 3408 | Advanced | 0.04 |  |  |
| 132 | intersectional*.mp. | 12416 | Advanced | 0.20 |  |  |
| 133 | (intersecting adj5 identit*).mp. | 1227 | Advanced | 0.60 |  |  |
| 134 | (interlocking adj3 system?).mp. | 206 | Advanced | 0.12 |  |  |
| 135 | (interlocking adj3 oppression?).mp. | 132 | Advanced | 0.30 |  |  |
| 136 | (overlapping adj5 identit*).mp. | 176 | Advanced | 0.19 |  |  |
| 137 | (overlapping adj2 social categor*).mp. | 1 | Advanced | 0.76 |  |  |
| 138 | (multiple adj2 social identit*).mp. | 250 | Advanced | 0.35 |  |  |
| 139 | (intersecting adj2 social categor*).mp. | 19 | Advanced | 0.27 |  |  |
| 140 | (multiple adj3 vulnerabilit*).mp. | 248 | Advanced | 0.34 |  |  |
| 141 | (intersecting adj3 vulnerabilit*).mp. | 46 | Advanced | 0.06 |  |  |
| 142 | (multiple adj3 intersection*).mp. | 542 | Advanced | 0.20 |  |  |
| 143 | (layered adj3 disadvantage?).mp. | 1 | Advanced | 0.34 |  |  |
| 144 | (complex adj2 social disadvantage?).mp. | 1 | Advanced | 0.32 |  |  |
| 145 | (Multidimensional adj3 inequalit*).mp. | 43 | Advanced | 0.39 |  |  |
| 146 | (Multi-dimensional adj3 inequalit*).mp. | 3 | Advanced | 0.26 |  |  |
| 147 | (Multifactorial adj3 disadvantage?).mp. | 1 | Advanced | 0.08 |  |  |
| 148 | (Multi-factorial adj3 disadvantage?).mp. | 0 | Advanced | 0.19 |  |  |
| 149 | (Compound adj3 disadvantage?).mp. | 26 | Advanced | 0.10 |  |  |
| 150 | (Cumulative adj3 disadvantage?).mp. | 421 | Advanced | 0.13 |  |  |
| 151 | (intersection between adj2 (race or racial or ethnic*)).mp. | 48 | Advanced | 1.23 |  |  |
| 152 | (social adj2 power).mp. | 3081 | Advanced | 0.36 |  |  |
| 153 | (interpersonal adj2 power).mp. | 422 | Advanced | 0.33 |  |  |
| 154 | (relational adj2 power).mp. | 250 | Advanced | 0.21 |  |  |
| 155 | (structural adj2 inequalit*).mp. | 1152 | Advanced | 0.08 |  |  |
| 156 | (systemic adj2 (inequalit* or inequit*)).mp. | 765 | Advanced | 0.21 |  |  |
| 157 | (institutional adj2 inequalit*).mp. | 80 | Advanced | 0.12 |  |  |
| 158 | (embedded adj2 inequalit*).mp. | 61 | Advanced | 0.16 |  |  |
| 159 | or/131-158 | 19931 | Advanced | 0.04 |  |  |
| 160 | 130 and 159 | 920 | Advanced | 0.02 |  |  |
| 161 | limit 160 to english language | 871 | Advanced | 0.10 |  |  |
| 162 | remove duplicates from 161 | 869 | Advanced | 5.03 |  |  |

EBM Reviews - Cochrane Central Register of Controlled Trials

| **#** | **Searches** | **Results** | **Type** | **Runtime** |  |  |
| --- | --- | --- | --- | --- | --- | --- |
|  | | | | | | |
| 1 | exp Artificial Intelligence/ | 3800 | Advanced | 0.01 |  |  |
| 2 | Big Data/ | 26 | Advanced | 0.01 |  |  |
| 3 | Biomedical Technology/ | 57 | Advanced | 0.00 |  |  |
| 4 | Computer Heuristics/ | 1 | Advanced | 0.00 |  |  |
| 5 | Computer-Aided Design/ | 495 | Advanced | 0.00 |  |  |
| 6 | Data Aggregation/ | 0 | Advanced | 0.00 |  |  |
| 7 | Data Analysis/ | 163 | Advanced | 0.00 |  |  |
| 8 | Data Mining/ | 61 | Advanced | 0.00 |  |  |
| 9 | Data Science/ | 4 | Advanced | 0.00 |  |  |
| 10 | Decision Making, Computer-Assisted/ | 164 | Advanced | 0.00 |  |  |
| 11 | Decision Trees/ | 332 | Advanced | 0.00 |  |  |
| 12 | Deep Learning/ | 388 | Advanced | 0.00 |  |  |
| 13 | exp Automation/ | 2643 | Advanced | 0.01 |  |  |
| 14 | Expert Systems/ | 75 | Advanced | 0.00 |  |  |
| 15 | Image Interpretation, Computer-Assisted/ | 1095 | Advanced | 0.00 |  |  |
| 16 | Image Processing, Computer-Assisted/ | 2345 | Advanced | 0.00 |  |  |
| 17 | Machine Learning/ | 782 | Advanced | 0.00 |  |  |
| 18 | Neural Networks, Computer/ | 360 | Advanced | 0.00 |  |  |
| 19 | Numerical Analysis, Computer-Assisted/ | 34 | Advanced | 0.00 |  |  |
| 20 | Pattern Recognition, Automated/ | 240 | Advanced | 0.00 |  |  |
| 21 | Radiographic Image Interpretation, Computer-Assisted/ | 618 | Advanced | 0.00 |  |  |
| 22 | Signal Processing, Computer-Assisted/ | 940 | Advanced | 0.00 |  |  |
| 23 | exp Supervised Machine Learning/ | 94 | Advanced | 0.01 |  |  |
| 24 | Support Vector Machine/ | 74 | Advanced | 0.00 |  |  |
| 25 | Unsupervised Machine Learning/ | 8 | Advanced | 0.00 |  |  |
| 26 | "artif#cial intelligence".mp. | 3547 | Advanced | 0.06 |  |  |
| 27 | "big data".mp. | 231 | Advanced | 0.05 |  |  |
| 28 | ("AI" and intelligence).tw. | 1687 | Advanced | 0.00 |  |  |
| 29 | ((neural* adj2 network*) and computer).mp. | 658 | Advanced | 0.09 |  |  |
| 30 | ((text? or textual*) adj2 analy*).mp. | 176 | Advanced | 0.26 |  |  |
| 31 | (algor#th* adj3 (comput* or machine? or intelligen* or art#ficial* or analy*)).mp. | 3129 | Advanced | 0.33 |  |  |
| 32 | (art#ficial* adj2 intelligen*).mp. | 3591 | Advanced | 0.00 |  |  |
| 33 | (automat* adj2 algor#th*).mp. | 493 | Advanced | 0.04 |  |  |
| 34 | (automat* adj2 pattern? adj2 analy*).mp. | 1 | Advanced | 0.07 |  |  |
| 35 | (automat* adj2 pattern? adj2 recog*).mp. | 242 | Advanced | 0.04 |  |  |
| 36 | (automat* adj2 pattern? adj2 classifi*).mp. | 1 | Advanced | 0.07 |  |  |
| 37 | (automat*4 and (computer* or technolog*)).mp. | 4335 | Advanced | 0.09 |  |  |
| 38 | (comput* adj1 analy*).mp. | 1838 | Advanced | 0.06 |  |  |
| 39 | (comput* adj2 knowledge adj2 (acquisition or representation*)).mp. | 0 | Advanced | 0.06 |  |  |
| 40 | (comput* adj2 pattern? adj2 analy*).mp. | 2 | Advanced | 0.04 |  |  |
| 41 | (comput* adj2 pattern? adj2 recog*).mp. | 7 | Advanced | 0.01 |  |  |
| 42 | (comput* adj2 pattern? adj2 classifi*).mp. | 0 | Advanced | 0.01 |  |  |
| 43 | (comput* adj2 reasoning*).mp. | 12 | Advanced | 0.03 |  |  |
| 44 | (computat* adj2 intelligen*).mp. | 8 | Advanced | 0.01 |  |  |
| 45 | (computational adj1 approach??).mp. | 37 | Advanced | 0.05 |  |  |
| 46 | (computational adj1 repurpos*).mp. | 0 | Advanced | 0.01 |  |  |
| 47 | (computer* adj2 vision*).mp. | 331 | Advanced | 0.04 |  |  |
| 48 | (computer* adj2 heuristic*).mp. | 4 | Advanced | 0.02 |  |  |
| 49 | (data adj1 mining).mp. | 377 | Advanced | 0.04 |  |  |
| 50 | (deep* adj2 learn*).mp. | 1476 | Advanced | 0.10 |  |  |
| 51 | (deep* adj2 network*).mp. | 350 | Advanced | 0.00 |  |  |
| 52 | (digital adj2 technolog*).mp. | 1056 | Advanced | 0.01 |  |  |
| 53 | (hierarchic* adj1 learn*).mp. | 10 | Advanced | 0.02 |  |  |
| 54 | (inductive* adj1 machine* adj1 learn*).mp. | 0 | Advanced | 0.02 |  |  |
| 55 | (information?? adj1 technolog*).mp. | 1045 | Advanced | 0.04 |  |  |
| 56 | (knowledge acqui* and computer*).mp. | 96 | Advanced | 0.04 |  |  |
| 57 | (knowledge discovery and computer).mp. | 4 | Advanced | 0.02 |  |  |
| 58 | (knowledge represent* and computer).mp. | 2 | Advanced | 0.04 |  |  |
| 59 | (learning adj1 model* adj1 algorithm*).mp. | 3 | Advanced | 0.11 |  |  |
| 60 | (learning adj2 algorithm*).mp. | 1104 | Advanced | 0.01 |  |  |
| 61 | (learning adj3 labeled data).mp. | 0 | Advanced | 0.03 |  |  |
| 62 | ((logistic* or logit) adj2 regress*).mp. | 28976 | Advanced | 0.10 |  |  |
| 63 | (machine? adj2 assist* adj2 discover*).mp. | 0 | Advanced | 0.09 |  |  |
| 64 | (machine? adj2 intelligen*).mp. | 64 | Advanced | 0.00 |  |  |
| 65 | (machine? adj2 learn*).mp. | 3604 | Advanced | 0.01 |  |  |
| 66 | (ML adj2 (framework? or tool*)).mp. | 19 | Advanced | 0.11 |  |  |
| 67 | ML algorithm*.mp. | 183 | Advanced | 0.01 |  |  |
| 68 | ML application*.mp. | 15 | Advanced | 0.06 |  |  |
| 69 | ML method*.mp. | 186 | Advanced | 0.21 |  |  |
| 70 | MLP.mp. | 163 | Advanced | 0.01 |  |  |
| 71 | (native adj2 bayes).mp. | 1 | Advanced | 0.03 |  |  |
| 72 | (learn* adj2 bayes).mp. | 3 | Advanced | 0.01 |  |  |
| 73 | (neural* adj2 network* adj2 (model* or comput*)).mp. | 516 | Advanced | 0.05 |  |  |
| 74 | (predictive adj2 algor#th*).mp. | 235 | Advanced | 0.03 |  |  |
| 75 | (predictive adj2 analy*).mp. | 818 | Advanced | 0.04 |  |  |
| 76 | (random* adj2 forest?).mp. | 1073 | Advanced | 0.47 |  |  |
| 77 | (regulari* adj3 algorithm*).mp. | 7 | Advanced | 0.01 |  |  |
| 78 | (regress* adj2 tree?).mp. | 314 | Advanced | 0.02 |  |  |
| 79 | (ridge adj2 regress*).mp. | 43 | Advanced | 0.01 |  |  |
| 80 | RVM.mp. | 47 | Advanced | 0.01 |  |  |
| 81 | (structural equation* adj2 model*).mp. | 1372 | Advanced | 0.05 |  |  |
| 82 | (support adj2 vector).mp. | 632 | Advanced | 0.03 |  |  |
| 83 | (vector? adj2 machine?).mp. | 620 | Advanced | 0.01 |  |  |
| 84 | (vector? adj2 network*).mp. | 9 | Advanced | 0.00 |  |  |
| 85 | adaptive algorithm*.mp. | 71 | Advanced | 0.01 |  |  |
| 86 | art#ficial neural network*.mp. | 403 | Advanced | 0.02 |  |  |
| 87 | artificial intelligence*.mp. | 3554 | Advanced | 0.02 |  |  |
| 88 | Bayesian network*.mp. | 197 | Advanced | 0.01 |  |  |
| 89 | big health* data.mp. | 0 | Advanced | 0.23 |  |  |
| 90 | chatgpt*.mp. | 221 | Advanced | 0.01 |  |  |
| 91 | (classification? adj2 algorithm*).mp. | 221 | Advanced | 0.02 |  |  |
| 92 | clustering algorithm*.mp. | 75 | Advanced | 0.01 |  |  |
| 93 | compression algorithm*.mp. | 7 | Advanced | 0.01 |  |  |
| 94 | computational intelligence.mp. | 5 | Advanced | 0.00 |  |  |
| 95 | computer cellular neural network*.mp. | 0 | Advanced | 0.02 |  |  |
| 96 | computer reasoning.mp. | 0 | Advanced | 0.01 |  |  |
| 97 | computer vision system?.mp. | 5 | Advanced | 0.08 |  |  |
| 98 | convolutional neural network*.mp. | 600 | Advanced | 0.02 |  |  |
| 99 | (crossvalidation or cross-validation).mp. | 1338 | Advanced | 0.05 |  |  |
| 100 | data analytic?.mp. | 147 | Advanced | 0.02 |  |  |
| 101 | data driven science?.mp. | 2 | Advanced | 0.05 |  |  |
| 102 | data-driven science?.mp. | 2 | Advanced | 0.02 |  |  |
| 103 | deep learning.mp. | 1406 | Advanced | 0.01 |  |  |
| 104 | detection algorithm*.mp. | 163 | Advanced | 0.01 |  |  |
| 105 | digital intervention?.mp. | 1279 | Advanced | 0.23 |  |  |
| 106 | ensemble learning*.mp. | 26 | Advanced | 0.03 |  |  |
| 107 | extreme learning machine*.mp. | 16 | Advanced | 0.02 |  |  |
| 108 | federated learning*.mp. | 8 | Advanced | 0.01 |  |  |
| 109 | feedforward neural network*.mp. | 13 | Advanced | 0.01 |  |  |
| 110 | generative ai*.mp. | 139 | Advanced | 0.16 |  |  |
| 111 | generative artificial intelligence*.mp. | 215 | Advanced | 0.01 |  |  |
| 112 | graph neural network*.mp. | 5 | Advanced | 0.02 |  |  |
| 113 | hierarchical learning.mp. | 5 | Advanced | 0.01 |  |  |
| 114 | hopfield neural network*.mp. | 0 | Advanced | 0.00 |  |  |
| 115 | image recognition.mp. | 63 | Advanced | 0.01 |  |  |
| 116 | inductive machine learning.mp. | 0 | Advanced | 0.01 |  |  |
| 117 | intelligent machine?.mp. | 1 | Advanced | 0.00 |  |  |
| 118 | intelligent system*.mp. | 39 | Advanced | 0.09 |  |  |
| 119 | machine assisted discovery.mp. | 0 | Advanced | 0.01 |  |  |
| 120 | (machine adj2 intelligence).mp. | 57 | Advanced | 0.01 |  |  |
| 121 | machine learning algorithm*.mp. | 629 | Advanced | 0.01 |  |  |
| 122 | machine learning.mp. | 3569 | Advanced | 0.01 |  |  |
| 123 | machine learnt.mp. | 0 | Advanced | 0.00 |  |  |
| 124 | machine prediction method*.mp. | 0 | Advanced | 0.04 |  |  |
| 125 | machine-assist* discover*.mp. | 0 | Advanced | 0.01 |  |  |
| 126 | multiple-instance learning algorithm*.mp. | 0 | Advanced | 0.03 |  |  |
| 127 | pattern analysis machine*.mp. | 0 | Advanced | 0.02 |  |  |
| 128 | predictive learning model*.mp. | 2 | Advanced | 0.02 |  |  |
| 129 | recurrent neural network*.mp. | 40 | Advanced | 0.01 |  |  |
| 130 | reinforcement machine learning*.mp. | 3 | Advanced | 0.01 |  |  |
| 131 | representation machine learning*.mp. | 0 | Advanced | 0.01 |  |  |
| 132 | self-learning.mp. | 348 | Advanced | 0.03 |  |  |
| 133 | (semisupervised adj2 (learn* or algorithm*)).mp. | 3 | Advanced | 0.02 |  |  |
| 134 | (semi-supervised adj2 (learn* or algorithm*)).mp. | 17 | Advanced | 0.03 |  |  |
| 135 | supervised machine* learn*.mp. | 134 | Advanced | 0.01 |  |  |
| 136 | support vector machine?.mp. | 614 | Advanced | 0.01 |  |  |
| 137 | support vector network?.mp. | 0 | Advanced | 0.02 |  |  |
| 138 | (train adj2 (dataset or data-set)).mp. | 9 | Advanced | 0.04 |  |  |
| 139 | (train adj2 algorithm*).mp. | 25 | Advanced | 0.00 |  |  |
| 140 | transfer learning.mp. | 91 | Advanced | 0.02 |  |  |
| 141 | transfer machine learning*.mp. | 0 | Advanced | 0.01 |  |  |
| 142 | (unsupervised adj2 (learn* or algorithm*)).mp. | 88 | Advanced | 0.03 |  |  |
| 143 | (un-supervised adj2 (learn* or algorithm*)).mp. | 0 | Advanced | 0.03 |  |  |
| 144 | or/1-143 | 60294 | Advanced | 0.03 |  |  |
| 145 | intersectional framework/ | 0 | Advanced | 0.00 |  |  |
| 146 | intersectional*.mp. | 132 | Advanced | 0.01 |  |  |
| 147 | (intersecting adj5 identit*).mp. | 5 | Advanced | 0.02 |  |  |
| 148 | (interlocking adj3 system?).mp. | 11 | Advanced | 0.02 |  |  |
| 149 | (interlocking adj3 oppression?).mp. | 0 | Advanced | 0.02 |  |  |
| 150 | (overlapping adj5 identit*).mp. | 0 | Advanced | 0.01 |  |  |
| 151 | (overlapping adj2 social categor*).mp. | 0 | Advanced | 0.06 |  |  |
| 152 | (multiple adj2 social identit*).mp. | 2 | Advanced | 0.01 |  |  |
| 153 | (intersecting adj2 social categor*).mp. | 0 | Advanced | 0.01 |  |  |
| 154 | (multiple adj3 vulnerabilit*).mp. | 9 | Advanced | 0.02 |  |  |
| 155 | (intersecting adj3 vulnerabilit*).mp. | 0 | Advanced | 0.00 |  |  |
| 156 | (multiple adj3 intersection*).mp. | 7 | Advanced | 0.02 |  |  |
| 157 | (layered adj3 disadvantage?).mp. | 0 | Advanced | 0.02 |  |  |
| 158 | (complex adj2 social disadvantage?).mp. | 0 | Advanced | 0.02 |  |  |
| 159 | (Multidimensional adj3 inequalit*).mp. | 0 | Advanced | 0.02 |  |  |
| 160 | (Multi-dimensional adj3 inequalit*).mp. | 0 | Advanced | 0.02 |  |  |
| 161 | (Multifactorial adj3 disadvantage?).mp. | 0 | Advanced | 0.00 |  |  |
| 162 | (Multi-factorial adj3 disadvantage?).mp. | 0 | Advanced | 0.02 |  |  |
| 163 | (Compound adj3 disadvantage?).mp. | 2 | Advanced | 0.01 |  |  |
| 164 | (Cumulative adj3 disadvantage?).mp. | 4 | Advanced | 0.01 |  |  |
| 165 | (intersection between adj2 (race or racial or ethnic*)).mp. | 0 | Advanced | 0.09 |  |  |
| 166 | (social adj2 power).mp. | 49 | Advanced | 0.03 |  |  |
| 167 | (interpersonal adj2 power).mp. | 3 | Advanced | 0.01 |  |  |
| 168 | (relational adj2 power).mp. | 1 | Advanced | 0.01 |  |  |
| 169 | (structural adj2 inequalit*).mp. | 7 | Advanced | 0.00 |  |  |
| 170 | (systemic adj2 (inequalit* or inequit*)).mp. | 13 | Advanced | 0.01 |  |  |
| 171 | (institutional adj2 inequalit*).mp. | 0 | Advanced | 0.01 |  |  |
| 172 | (embedded adj2 inequalit*).mp. | 0 | Advanced | 0.01 |  |  |
| 173 | or/145-172 | 237 | Advanced | 0.00 |  |  |
| 174 | 144 and 173 | 12 | Advanced | 0.00 |  |  |
| 175 | remove duplicates from 174 | 12 | Advanced | 0.07 |  |  |

EBM Reviews - Cochrane Database of Systematic Reviews <2005 to January 22, 2026>

| **#** | **Searches** | **Results** | **Type** | **Runtime** |  |  |
| --- | --- | --- | --- | --- | --- | --- |
|  | | | | | |  |
| 1 | "artif#cial intelligence".ti,ab. | 9 | Advanced | 0.04 |  |  |
| 2 | "big data".ti,ab. | 1 | Advanced | 0.02 |  |  |
| 3 | ("AI" and intelligence).tw. | 50 | Advanced | 0.01 |  |  |
| 4 | ((neural* adj2 network*) and computer).ti,ab. | 0 | Advanced | 0.01 |  |  |
| 5 | ((text? or textual*) adj2 analy*).ti,ab. | 5 | Advanced | 0.03 |  |  |
| 6 | (algor#th* adj3 (comput* or machine? or intelligen* or art#ficial* or analy*)).ti,ab. | 4 | Advanced | 0.04 |  |  |
| 7 | (art#ficial* adj2 intelligen*).ti,ab. | 9 | Advanced | 0.00 |  |  |
| 8 | (automat* adj2 algor#th*).ti,ab. | 2 | Advanced | 0.01 |  |  |
| 9 | (automat* adj2 pattern? adj2 analy*).ti,ab. | 0 | Advanced | 0.01 |  |  |
| 10 | (automat* adj2 pattern? adj2 recog*).ti,ab. | 0 | Advanced | 0.01 |  |  |
| 11 | (automat* adj2 pattern? adj2 classifi*).ti,ab. | 0 | Advanced | 0.01 |  |  |
| 12 | (automat*4 and (computer* or technolog*)).ti,ab. | 10 | Advanced | 0.01 |  |  |
| 13 | (comput* adj1 analy*).ti,ab. | 0 | Advanced | 0.00 |  |  |
| 14 | (comput* adj2 knowledge adj2 (acquisition or representation*)).ti,ab. | 0 | Advanced | 0.02 |  |  |
| 15 | (comput* adj2 pattern? adj2 analy*).ti,ab. | 0 | Advanced | 0.00 |  |  |
| 16 | (comput* adj2 pattern? adj2 recog*).ti,ab. | 0 | Advanced | 0.00 |  |  |
| 17 | (comput* adj2 pattern? adj2 classifi*).ti,ab. | 0 | Advanced | 0.00 |  |  |
| 18 | (comput* adj2 reasoning*).ti,ab. | 0 | Advanced | 0.00 |  |  |
| 19 | (computat* adj2 intelligen*).ti,ab. | 0 | Advanced | 0.00 |  |  |
| 20 | (computational adj1 approach??).ti,ab. | 0 | Advanced | 0.01 |  |  |
| 21 | (computational adj1 repurpos*).ti,ab. | 0 | Advanced | 0.00 |  |  |
| 22 | (computer* adj2 vision*).ti,ab. | 0 | Advanced | 0.01 |  |  |
| 23 | (computer* adj2 heuristic*).ti,ab. | 0 | Advanced | 0.00 |  |  |
| 24 | (data adj1 mining).ti,ab. | 0 | Advanced | 0.00 |  |  |
| 25 | (deep* adj2 learn*).ti,ab. | 1 | Advanced | 0.01 |  |  |
| 26 | (deep* adj2 network*).ti,ab. | 1 | Advanced | 0.00 |  |  |
| 27 | (digital adj2 technolog*).ti,ab. | 9 | Advanced | 0.01 |  |  |
| 28 | (hierarchic* adj1 learn*).ti,ab. | 0 | Advanced | 0.00 |  |  |
| 29 | (inductive* adj1 machine* adj1 learn*).ti,ab. | 0 | Advanced | 0.00 |  |  |
| 30 | (information?? adj1 technolog*).ti,ab. | 11 | Advanced | 0.01 |  |  |
| 31 | (knowledge acqui* and computer*).ti,ab. | 0 | Advanced | 0.01 |  |  |
| 32 | (knowledge discovery and computer).ti,ab. | 0 | Advanced | 0.01 |  |  |
| 33 | (knowledge represent* and computer).ti,ab. | 0 | Advanced | 0.00 |  |  |
| 34 | (learning adj1 model* adj1 algorithm*).ti,ab. | 0 | Advanced | 0.01 |  |  |
| 35 | (learning adj2 algorithm*).ti,ab. | 0 | Advanced | 0.00 |  |  |
| 36 | (learning adj3 labeled data).ti,ab. | 0 | Advanced | 0.01 |  |  |
| 37 | ((logistic* or logit) adj2 regress*).ti,ab. | 16 | Advanced | 0.01 |  |  |
| 38 | (machine? adj2 assist* adj2 discover*).ti,ab. | 0 | Advanced | 0.00 |  |  |
| 39 | (machine? adj2 intelligen*).ti,ab. | 0 | Advanced | 0.00 |  |  |
| 40 | (machine? adj2 learn*).ti,ab. | 3 | Advanced | 0.00 |  |  |
| 41 | (ML adj2 (framework? or tool*)).ti,ab. | 0 | Advanced | 0.02 |  |  |
| 42 | ML algorithm*.ti,ab. | 0 | Advanced | 0.00 |  |  |
| 43 | ML application*.ti,ab. | 0 | Advanced | 0.00 |  |  |
| 44 | ML method*.ti,ab. | 0 | Advanced | 0.01 |  |  |
| 45 | MLP.ti,ab. | 0 | Advanced | 0.00 |  |  |
| 46 | (native adj2 bayes).ti,ab. | 0 | Advanced | 0.01 |  |  |
| 47 | (learn* adj2 bayes).ti,ab. | 0 | Advanced | 0.00 |  |  |
| 48 | (neural* adj2 network* adj2 (model* or comput*)).ti,ab. | 0 | Advanced | 0.00 |  |  |
| 49 | (predictive adj2 algor#th*).ti,ab. | 0 | Advanced | 0.01 |  |  |
| 50 | (predictive adj2 analy*).ti,ab. | 3 | Advanced | 0.00 |  |  |
| 51 | (random* adj2 forest?).ti,ab. | 1 | Advanced | 0.01 |  |  |
| 52 | (regulari* adj3 algorithm*).ti,ab. | 0 | Advanced | 0.00 |  |  |
| 53 | (regress* adj2 tree?).ti,ab. | 0 | Advanced | 0.01 |  |  |
| 54 | (ridge adj2 regress*).ti,ab. | 0 | Advanced | 0.00 |  |  |
| 55 | RVM.ti,ab. | 0 | Advanced | 0.00 |  |  |
| 56 | (structural equation* adj2 model*).ti,ab. | 0 | Advanced | 0.01 |  |  |
| 57 | (support adj2 vector).ti,ab. | 2 | Advanced | 0.01 |  |  |
| 58 | (vector? adj2 machine?).ti,ab. | 2 | Advanced | 0.00 |  |  |
| 59 | (vector? adj2 network*).ti,ab. | 0 | Advanced | 0.00 |  |  |
| 60 | adaptive algorithm*.ti,ab. | 0 | Advanced | 0.00 |  |  |
| 61 | art#ficial neural network*.ti,ab. | 0 | Advanced | 0.00 |  |  |
| 62 | artificial intelligence*.ti,ab. | 9 | Advanced | 0.01 |  |  |
| 63 | Bayesian network*.ti,ab. | 3 | Advanced | 0.00 |  |  |
| 64 | big health* data.ti,ab. | 0 | Advanced | 0.01 |  |  |
| 65 | chatgpt*.ti,ab. | 0 | Advanced | 0.00 |  |  |
| 66 | (classification? adj2 algorithm*).ti,ab. | 1 | Advanced | 0.00 |  |  |
| 67 | clustering algorithm*.ti,ab. | 0 | Advanced | 0.01 |  |  |
| 68 | compression algorithm*.ti,ab. | 0 | Advanced | 0.00 |  |  |
| 69 | computational intelligence.ti,ab. | 0 | Advanced | 0.00 |  |  |
| 70 | computer cellular neural network*.ti,ab. | 0 | Advanced | 0.01 |  |  |
| 71 | computer reasoning.ti,ab. | 0 | Advanced | 0.00 |  |  |
| 72 | computer vision system?.ti,ab. | 0 | Advanced | 0.01 |  |  |
| 73 | convolutional neural network*.ti,ab. | 0 | Advanced | 0.00 |  |  |
| 74 | (crossvalidation or cross-validation).ti,ab. | 0 | Advanced | 0.01 |  |  |
| 75 | data analytic?.ti,ab. | 0 | Advanced | 0.01 |  |  |
| 76 | data driven science?.ti,ab. | 0 | Advanced | 0.01 |  |  |
| 77 | data-driven science?.ti,ab. | 0 | Advanced | 0.00 |  |  |
| 78 | deep learning.ti,ab. | 1 | Advanced | 0.00 |  |  |
| 79 | detection algorithm*.ti,ab. | 1 | Advanced | 0.01 |  |  |
| 80 | digital intervention?.ti,ab. | 10 | Advanced | 0.02 |  |  |
| 81 | ensemble learning*.ti,ab. | 0 | Advanced | 0.00 |  |  |
| 82 | extreme learning machine*.ti,ab. | 0 | Advanced | 0.01 |  |  |
| 83 | federated learning*.ti,ab. | 0 | Advanced | 0.00 |  |  |
| 84 | feedforward neural network*.ti,ab. | 0 | Advanced | 0.00 |  |  |
| 85 | generative ai*.ti,ab. | 0 | Advanced | 0.03 |  |  |
| 86 | generative artificial intelligence*.ti,ab. | 0 | Advanced | 0.00 |  |  |
| 87 | graph neural network*.ti,ab. | 0 | Advanced | 0.01 |  |  |
| 88 | hierarchical learning.ti,ab. | 0 | Advanced | 0.00 |  |  |
| 89 | hopfield neural network*.ti,ab. | 0 | Advanced | 0.00 |  |  |
| 90 | image recognition.ti,ab. | 1 | Advanced | 0.01 |  |  |
| 91 | inductive machine learning.ti,ab. | 0 | Advanced | 0.00 |  |  |
| 92 | intelligent machine?.ti,ab. | 0 | Advanced | 0.00 |  |  |
| 93 | intelligent system*.ti,ab. | 0 | Advanced | 0.01 |  |  |
| 94 | machine assisted discovery.ti,ab. | 0 | Advanced | 0.00 |  |  |
| 95 | (machine adj2 intelligence).ti,ab. | 0 | Advanced | 0.00 |  |  |
| 96 | machine learning algorithm*.ti,ab. | 0 | Advanced | 0.00 |  |  |
| 97 | machine learning.ti,ab. | 3 | Advanced | 0.00 |  |  |
| 98 | machine learnt.ti,ab. | 0 | Advanced | 0.00 |  |  |
| 99 | machine prediction method*.ti,ab. | 0 | Advanced | 0.00 |  |  |
| 100 | machine-assist* discover*.ti,ab. | 0 | Advanced | 0.00 |  |  |
| 101 | multiple-instance learning algorithm*.ti,ab. | 0 | Advanced | 0.01 |  |  |
| 102 | pattern analysis machine*.ti,ab. | 0 | Advanced | 0.00 |  |  |
| 103 | predictive learning model*.ti,ab. | 0 | Advanced | 0.00 |  |  |
| 104 | recurrent neural network*.ti,ab. | 0 | Advanced | 0.01 |  |  |
| 105 | reinforcement machine learning*.ti,ab. | 0 | Advanced | 0.00 |  |  |
| 106 | representation machine learning*.ti,ab. | 0 | Advanced | 0.00 |  |  |
| 107 | self-learning.ti,ab. | 0 | Advanced | 0.01 |  |  |
| 108 | (semisupervised adj2 (learn* or algorithm*)).ti,ab. | 0 | Advanced | 0.00 |  |  |
| 109 | (semi-supervised adj2 (learn* or algorithm*)).ti,ab. | 0 | Advanced | 0.01 |  |  |
| 110 | supervised machine* learn*.ti,ab. | 0 | Advanced | 0.00 |  |  |
| 111 | support vector machine?.ti,ab. | 2 | Advanced | 0.00 |  |  |
| 112 | support vector network?.ti,ab. | 0 | Advanced | 0.01 |  |  |
| 113 | (train adj2 (dataset or data-set)).ti,ab. | 0 | Advanced | 0.01 |  |  |
| 114 | (train adj2 algorithm*).ti,ab. | 2 | Advanced | 0.00 |  |  |
| 115 | transfer learning.ti,ab. | 0 | Advanced | 0.01 |  |  |
| 116 | transfer machine learning*.ti,ab. | 0 | Advanced | 0.01 |  |  |
| 117 | (unsupervised adj2 (learn* or algorithm*)).ti,ab. | 0 | Advanced | 0.01 |  |  |
| 118 | (un-supervised adj2 (learn* or algorithm*)).ti,ab. | 0 | Advanced | 0.00 |  |  |
| 119 | or/1-118 | 121 | Advanced | 0.00 |  |  |
| 120 | intersectional*.ti,ab. | 1 | Advanced | 0.04 |  |  |
| 121 | (intersecting adj5 identit*).ti,ab. | 0 | Advanced | 0.08 |  |  |
| 122 | (interlocking adj3 system?).ti,ab. | 0 | Advanced | 0.13 |  |  |
| 123 | (interlocking adj3 oppression?).ti,ab. | 0 | Advanced | 0.04 |  |  |
| 124 | (overlapping adj5 identit*).ti,ab. | 0 | Advanced | 0.08 |  |  |
| 125 | (overlapping adj2 social categor*).ti,ab. | 0 | Advanced | 0.09 |  |  |
| 126 | (multiple adj2 social identit*).ti,ab. | 0 | Advanced | 0.08 |  |  |
| 127 | (intersecting adj2 social categor*).ti,ab. | 0 | Advanced | 0.07 |  |  |
| 128 | (multiple adj3 vulnerabilit*).ti,ab. | 0 | Advanced | 0.10 |  |  |
| 129 | (intersecting adj3 vulnerabilit*).ti,ab. | 0 | Advanced | 0.08 |  |  |
| 130 | (multiple adj3 intersection*).ti,ab. | 0 | Advanced | 0.09 |  |  |
| 131 | (layered adj3 disadvantage?).ti,ab. | 0 | Advanced | 0.07 |  |  |
| 132 | (complex adj2 social disadvantage?).ti,ab. | 0 | Advanced | 0.05 |  |  |
| 133 | (Multidimensional adj3 inequalit*).ti,ab. | 0 | Advanced | 0.06 |  |  |
| 134 | (Multi-dimensional adj3 inequalit*).ti,ab. | 0 | Advanced | 0.05 |  |  |
| 135 | (Multifactorial adj3 disadvantage?).ti,ab. | 0 | Advanced | 0.09 |  |  |
| 136 | (Multi-factorial adj3 disadvantage?).ti,ab. | 0 | Advanced | 0.09 |  |  |
| 137 | (Compound adj3 disadvantage?).ti,ab. | 0 | Advanced | 0.05 |  |  |
| 138 | (Cumulative adj3 disadvantage?).ti,ab. | 0 | Advanced | 0.04 |  |  |
| 139 | (intersection between adj2 (race or racial or ethnic*)).ti,ab. | 0 | Advanced | 0.12 |  |  |
| 140 | (social adj2 power).ti,ab. | 0 | Advanced | 0.05 |  |  |
| 141 | (interpersonal adj2 power).ti,ab. | 0 | Advanced | 0.04 |  |  |
| 142 | (relational adj2 power).ti,ab. | 0 | Advanced | 0.08 |  |  |
| 143 | (structural adj2 inequalit*).ti,ab. | 1 | Advanced | 0.14 |  |  |
| 144 | (systemic adj2 (inequalit* or inequit*)).ti,ab. | 0 | Advanced | 0.04 |  |  |
| 145 | (institutional adj2 inequalit*).ti,ab. | 0 | Advanced | 0.10 |  |  |
| 146 | (embedded adj2 inequalit*).ti,ab. | 0 | Advanced | 0.05 |  |  |
| 147 | or/120-146 | 2 | Advanced | 0.03 |  |  |
| 148 | 119 and 147 | 0 | Advanced | 0.10 |  |  |
|  |  |  |  |  |  |  |
